# Design of novel triply periodic minimal surface (TPMS) bone scaffold with multi-functional pores: lower stress shielding and higher mass transport capacity

**DOI:** 10.3389/fbioe.2024.1401899

**Published:** 2024-06-26

**Authors:** Jian Jiang, Yi Huo, Xing Peng, Chengwei Wu, Hanxing Zhu, Yongtao Lyu

**Affiliations:** ^1^ Department of Spinal Surgery, Central Hospital of Dalian University of Technology, Dalian, China; ^2^ School of Mechanics and Aerospace Engineering, Dalian University of Technology, Dalian, China; ^3^ Tribology Research Institute, School of Mechanical Engineering, Southwest Jiaotong University, Chengdu, China; ^4^ State Key Laboratory of Structural Analysis Optimization and CAE Software for Industrial Equipment, Department of Engineering Mechanics, Dalian University of Technology, Dalian, China; ^5^ School of Engineering, Cardiff University, Cardiff, United Kingdom

**Keywords:** bone scaffold, multi-functional pore, additive manufacturing, mechanical behavior, mass transport capacity

## Abstract

**Background:** The bone repair requires the bone scaffolds to meet various mechanical and biological requirements, which makes the design of bone scaffolds a challenging problem. Novel triply periodic minimal surface (TPMS)-based bone scaffolds were designed in this study to improve the mechanical and biological performances simultaneously.

**Methods:** The novel bone scaffolds were designed by adding optimization-guided multi-functional pores to the original scaffolds, and finite element (FE) method was used to evaluate the performances of the novel scaffolds. In addition, the novel scaffolds were fabricated by additive manufacturing (AM) and mechanical experiments were performed to evaluate the performances.

**Results:** The FE results demonstrated the improvement in performance: the elastic modulus reduced from 5.01 GPa (original scaffold) to 2.30 GPa (novel designed scaffold), resulting in lower stress shielding; the permeability increased from 8.58 × 10^−9^ m^2^ (original scaffold) to 5.14 × 10^−8^ m^2^ (novel designed scaffold), resulting in higher mass transport capacity.

**Conclusion:** In summary, the novel TPMS scaffolds with multi-functional pores simultaneously improve the mechanical and biological performances, making them ideal candidates for bone repair. Furthermore, the novel scaffolds expanded the design domain of TPMS-based bone scaffolds, providing a promising new method for the design of high-performance bone scaffolds.

## 1 Introduction

The demand for high performance artificial bone implants is growing due to the rising prevalence of bone diseases and traumas (Kurtz et al., 2007; Campana et al., 2014; Wallace et al., 2017). Porous bone scaffolds are considered ideal for bone repair because their porosity and stiffness can be adjusted to mimic human bone. Additionally, a microscopic porous scaffold can provide a suitable physiological environment for bone ingrowth (Peng et al., 2022). Recently, studies have used triply periodic minimal surface (TPMS) as a porous structure to design bone scaffold (Davoodi et al., 2020; Jiang et al., 2020; Vijayavenkataraman et al., 2020). The advantage of TPMS lies in its ability to easily adjust pore sizes using control equations, allowing the mechanical properties of the scaffolds to closely resemble those of human bone (Maconachie et al., 2019). Moreover, TPMS can provide micropores larger than 300.0 μm to allow for bone growth. Barba et al. (2019) concluded that a 300.0–600.0 μm pore size is best for osseointegration since it facilitates vascularization and cell growth. Consequently, TPMS-based bone scaffolds are being extensively studied and designed to address clinical challenges. On the other hand, additive manufacturing (AM) provides an ideal solution for manufacturing highly complex geometries such as TPMS scaffolds (Askari et al., 2020). AM describes a range of processes used to fabricate components directly from a digital representation of the intended geometry by the layer wise combination of a common source material (Zhai et al., 2014). As a result, TPMS-based bone scaffolds manufactured using AM have become the ideal choice for bone implants.

However, there are two problems with TPMS-based scaffolds that urgently need to be solved—the stress shielding effect caused by the high elastic modulus and the insufficient mass transport capacity caused by the low permeability. A bone scaffold should have an elastic modulus similar to that of the human bone to avoid the stress shielding effect. This effect occurs when the load is predominantly borne by the bone scaffold, leading to loosening at the interface (Barba et al., 2019). Although the elastic modulus of a TPMS scaffold is lower than that of the traditional cubic structure, it is still higher than that of cancellous bone. Sevilla et al. (2007) reported that the elastic modulus of cancellous bone is 1.08 GPa. Wu et al. (2018) investigated the elastic modulus of cancellous bone under different loading directions. The results indicated that the modulus of cancellous bone is 3.47 GPa in the longitudinal direction and 2.57 ± 0.28 GPa in the transverse direction. While there are discrepancies in reported elastic moduli of cancellous bone across studies, it is generally agreed that a bone scaffold’s elastic modulus should not exceed 3.00 GPa to align with cancellous bone. However, the modulus of a Schwarz P TPMS scaffold with 70% porosity is 5.60 GPa (Khaleghi et al., 2021), which is greater than that of cancellous bone. Additionally, the permeability of cancellous bone is in the range of 3.66 × 10^–8^ m^2^ to 1.90 × 10^–7^ m^2^ (Rabiatul et al., 2021), but the permeability of a TPMS structure is in the range of 4.31 × 10^–10^ m^2^ to 8.44 × 10^–9^ m^2^ (Santos et al., 2020). Therefore, broadening the limited TPMS design domain to simultaneously improve its mechanical and biological performances to meet the clinical needs is a big challenge in the design of TPMS bone scaffolds.

In recent years, there have been efforts to enhance the performance of TPMS scaffolds through innovative design approaches. The most common design method is structural optimization, which aims to find the best design scheme based on specific goals and constraints. Moreover, the integration of structural optimization with additive manufacturing (AM) presents a realm of creative opportunities for bone scaffold design (Tan et al., 2017). This method has been applied in clinic to fabricate devices such as pelvic protheses (Iqbal et al., 2019), craniofacial prostheses (Sutradhar et al., 2016), and femoral stem protheses (Arabnejad et al., 2017; Sun et al., 2018). Additionally, some studies have designed hybrid TPMS scaffolds and functional graded TPMS scaffolds to improve the performances. For example, Liu et al. (2022) designed a hybrid TPMS structure to increase the permeability to 1.20 × 10^–8^ m^2^. Fan et al. (2021) designed functional graded TPMS scaffolds to control the elastic modulus, but both the graded TPMS scaffolds and the hybrid TPMS scaffolds still have the elastic moduli much higher than that of cancellous bone. In addition, these designed scaffolds have many shortcomings, such as poor controllability and lack of reasonable optimization framework guidance. Take the design of functional graded TPMS bone scaffold as example, before designing a functional graded bone scaffold, scholars cannot predict its performance. In these studies, the scaffolds were designed first, and then the performance improvements of the new scaffolds can be proved only by the research results. Therefore, such design method is very inefficient. In addition, the design method in the current study usually can only consider one optimization objective such as elastic modulus, but the permeability is not evaluated. Besides, whether the designed TPMS bone scaffolds meet the requirements of the manufacturing technique has not been considered and explained. Therefore, novel scaffolds that can be controlled and simultaneously improve the mechanical and biological performances are needed to be designed.

In order to address the problems of poor controllability and limited optimization objectives in the optimal design of TPMS bone scaffolds, we propose a novel design method: introducing a new geometric variable, that is, optimization-guided multi-functional pore. The novel TPMS scaffolds with optimization-guided multi-functional pores were designed to address the problems of high elastic modulus and low permeability in this study. The functions of these pores are: to reduce the elastic modulus, to improve the permeability, and to broaden the design domain. Therefore, we name it “multi-functional pores” to represent these functions. In addition, the position of multi-function pore is not random, but determined under the guidance of optimization theory. After determining the position of the multi-function pore, change its radius to evaluate the effect of different size pores on the performance. The introduction of this multi-functional pore solves many difficulties in the design of TPMS bone scaffolds. First, the multi-functional pore is designed under the optimization framework of reducing the elastic modulus of the bone scaffold, and the elastic modulus of the new designed bone scaffold must be reduced to avoid the stress shielding effect. In the previous design methods, the performance of the new structure cannot be predicted before the design. Therefore, the proposed design method has higher controllability and efficiency. Second, there are few design variables of TPMS structure at present. By introducing the new variable of multi-function pore, the design domain of TPMS structure can be broadened, and the design of high-performance TPMS bone scaffold can be realized. To investigate the performances of the novel designed bone scaffolds, all the scaffolds were fabricated from Ti6Al4V using selective laser melting (SLM). Experiments and FE simulations were used to evaluate the elastic modulus, permeability, and anisotropy of the structures.

## 2 Materials and methods

In this section, the method to design the novel TPMS scaffolds with multi-functional pores is first detailed. Then, the experimental and simulation methods for evaluating the elastic modulus and permeability behaviors of the TPMS scaffolds are illustrated. In the end, the anisotropic elastic response of the new scaffold is evaluated using the numerical homogenization method.

### 2.1 Modelling method of basic TPMS scaffolds

**FIGURE 1:**
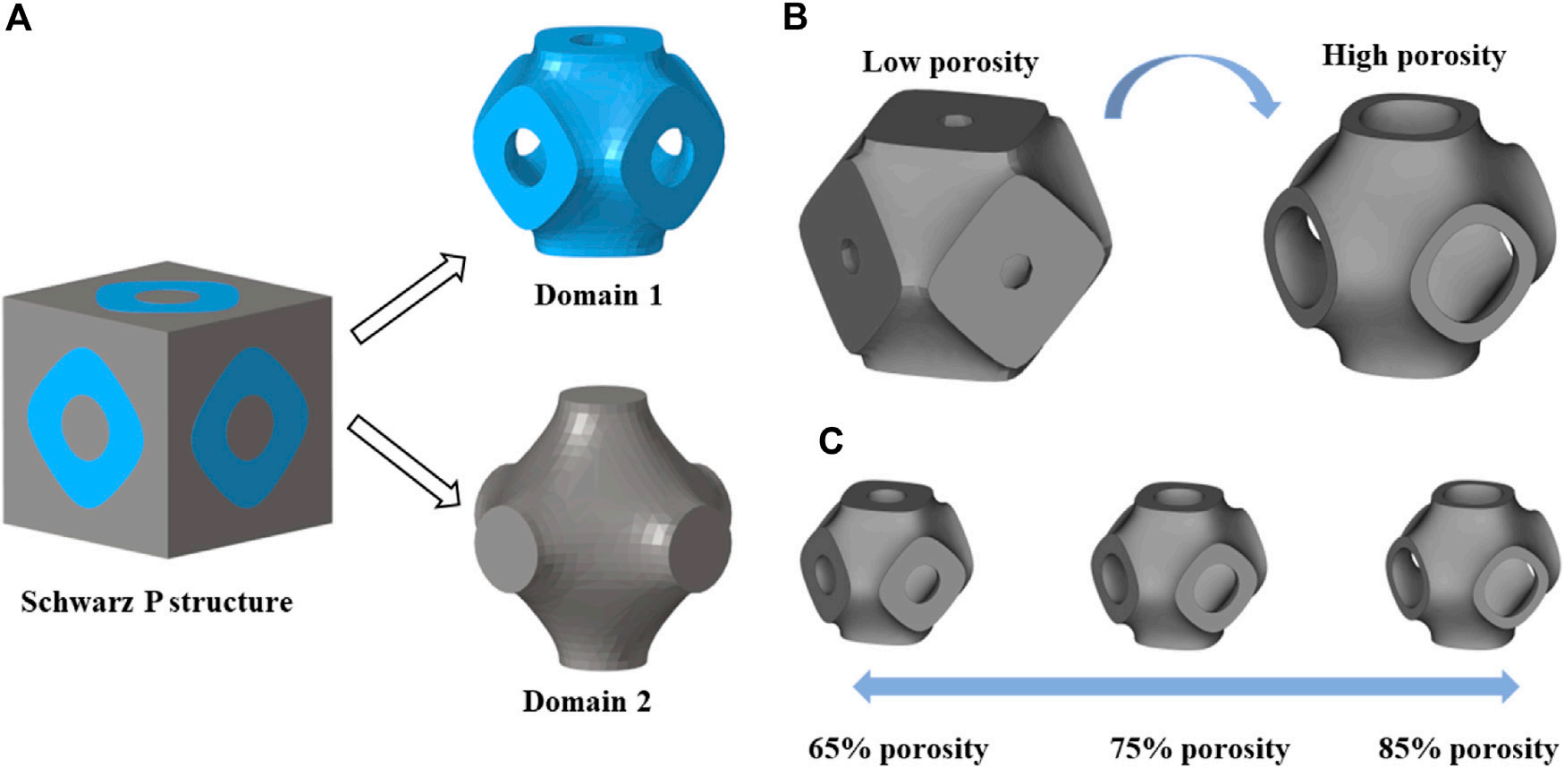
**(A)** Two domains divided by a Schwarz P structure. **(B)** Schwarz P structures with different porosities obtained by changing the constant c . **(C)** Three basic Schwarz P structures with different porosities (65%, 75%, and 85%).

TPMS is a minimal surface that can extend periodically in three directions, and its topological shape is determined by functional expressions. Common TPMS structures include Schwarz P, Gyroid, Diamond, I-WP, etc. (Blanquer et al., 2017). It is worth mentioning that the concept of minimal surface was first proposed by the scientist Schwarz in 1883 (Strömberg, 2021), so Schwarz P is also one of the most classical and widely used types of TPMS structures. Besides, this structure has been shown to have a more stable curvature to promote cell growth (Blanquer et al., 2017). Therefore, this paper chooses Schwarz P as the basis of structural design. The 3D Schwarz P structure was formed by adding the thickness of the minimal surface (Figure 1A). The Schwarz P structure can be characterized by the following mathematical function (Khaleghi et al., 2021):

$$f\left(x,y,x\right)=\cos\left(\frac{2\pi }{n}x\right)+\cos\left(\frac{2\pi }{n}y\right)+\cos\left(\frac{2\pi }{n}z\right)-c$$ (1)

where f determines the TPMS topology type; x,y,z are the coordinates of a point in the design space; n denotes the length of a unit cell, and the constant c is used to control the two-phase domain, which determines the porosity of the structure (Peng et al., 2023). With the increase of the constant c from −0.5 to 0.5, the porosity of the Schwarz P structure increases (Figure 1B). Previous studies have proved that there is a linear relationship between the constant c in the Eq. 1 and the porosity of TPMS structure (Al-Ketan and Abu Al-Rub, 2019). As for Schwarz P, the porosity can be represented by: 0.2876 c +0.4967. Therefore, when the constant c equal to 0.53, 0.88, and 1.23, the Schwarz P structure with porosity of 65%, 75% and 85% can be obtained (Figure 1C). These structures were visualized using in-house developed MATLAB code (R2020b, MathWorks, Massachusetts, US), and the dimensions of unit cell were set to 2.5 mm × 2.5 mm × 2.5 mm.

### 2.2 Design of the novel TPMS structures with multi-functional pores

By adjusting the constants in Eq. 1, only structures with different porosities can be obtained, that is, porosity is the only variable when designing the scaffolds. However, the mass transport capacity of the scaffold depends on the pore size and the obstructed area (Ali et al., 2020). Therefore, the multi-functional pores were added to the scaffold to improve the permeability. These multi-functional pores were designed and guided by structural optimization method, which is detailed in this section.

Mechanical parameters, including the elastic modulus and anisotropy, are crucial factors for structural design (Feng et al., 2021), and they can be obtained through the constitutive relation. The constitutive relation of a TPMS structure is given by Eq. 2 (Lee et al., 2017):

$$\left\{\sigma \right\}=\left[C\right]\left\{\varepsilon \right\}$$ (2)

where the stress and strain can be expressed in matrix form:

$$\left\{\sigma \right\}=\left(\begin{array}{c} \begin{array}{c} \sigma_{11}\\ \sigma_{22} \end{array}\\ \sigma_{33}\\ \sigma_{12}\\ \sigma_{13}\\ \sigma_{23} \end{array}\right)\;\left\{\varepsilon \right\}=\left(\begin{array}{c} \begin{array}{c} \varepsilon_{11}\\ \varepsilon_{22} \end{array}\\ \varepsilon_{33}\\ \varepsilon_{12}\\ \varepsilon_{13}\\ \varepsilon_{23} \end{array}\right)$$ (3)

The stiffness matrix can be expressed as Eq. 4:

$$\left[C\right]=\left[\begin{array}{cccccc} C_{11} & C_{12} & C_{13} & C_{14} & C_{15} & C_{16}\\ C_{21} & C_{22} & C_{23} & C_{24} & C_{25} & C_{26}\\ C_{31} & C_{32} & C_{33} & C_{34} & C_{35} & C_{36}\\ C_{41} & C_{42} & C_{43} & C_{44} & C_{45} & C_{46}\\ C_{51} & C_{52} & C_{53} & C_{54} & C_{55} & C_{56}\\ C_{61} & C_{62} & C_{63} & C_{64} & C_{65} & C_{66} \end{array}\right]$$ (4)

Moreover, Schwarz P is a cubic symmetric structure with three independent elastic constants, which means that $C_{11}=C_{22}=C_{33};\;C_{12}=C_{13}=C_{23};\;C_{44}=C_{55}=C_{66}$ ; and all other constants are zero. Thus, the stiffness matrix can be simplified as Eq. 5:

$$\left[C\right]=\left[\begin{array}{cccccc} C_{11} & C_{12} & C_{12} & 0 & 0 & 0\\ C_{12} & C_{11} & C_{12} & 0 & 0 & 0\\ C_{12} & C_{12} & C_{11} & 0 & 0 & 0\\ 0 & 0 & 0 & C_{44} & 0 & 0\\ 0 & 0 & 0 & 0 & C_{44} & 0\\ 0 & 0 & 0 & 0 & 0 & C_{44} \end{array}\right]$$ (5)

where $C_{11},C_{12}$ , and $C_{44}$ are the three independent elastic constants of the Schwarz P structure. Typically, when the two load cases $\left\{\varepsilon^{\left(1\right)}\right\}=\left\{1\;0\;0\;0\;0\;0\;\right\}$ and $\left\{\varepsilon^{\left(2\right)}\right\}=\left\{1\;1\;1\;0\;0\;0\;\right\}$ are applied to the Schwarz P structure, the relationship between $C_{ij}$ and $\sigma_{ij}$ can be determined based on Eqs 2, 3, 5:

$$\left\{\begin{array}{l} c_{11}=\sigma_{11}^{\left(1\right)}\\ c_{12}=\sigma_{22}^{\left(1\right)}=\sigma_{33}^{\left(1\right)}\\ c_{11}+2c_{12}=\sigma_{11}^{\left(2\right)}=\sigma_{22}^{\left(2\right)}=\sigma_{33}^{\left(2\right)} \end{array}\right.$$ (6)

The energies of two load cases can be defined as shown in Eq. 7 (Ma et al., 2021):

$$\left\{\begin{array}{l} w^{\left(1\right)}=\frac{1}{2}\sigma_{11}^{\left(1\right)}V\\ w^{\left(2\right)}=\frac{1}{2}\left(\sigma_{11}^{\left(2\right)}+\sigma_{22}^{\left(2\right)}+\sigma_{33}^{\left(2\right)}\right)V \end{array}\right.$$ (7)

where V represents the volume of the Schwarz P structure. Based on Eqs 6, 7, the relationship between the energies and elastic constants can be expressed as Eq. 8:

$$\left\{\begin{array}{l} c_{11}=\frac{2}{V}w^{\left(1\right)}\\ c_{12}=\frac{1}{V}\left[\frac{1}{3}w^{\left(2\right)}-w^{\left(1\right)}\right] \end{array}\right.$$ (8)

The elastic modulus of the Schwarz P structure can be obtained as Eq. 9 (Feng et al., 2021):

$$E=\frac{C_{11}^{2}+C_{12}C_{11}-2C_{12}{\;}^{2}}{C_{11}+C_{12}}$$ (9)

Based on Eqs 8, 9, the elastic modulus of the Schwarz P structure can be expressed as Eq. 10:

$$E=\frac{18w^{\left(1\right)}w^{\left(2\right)}-2{\left[w^{\left(2\right)}\right]}^{2}}{3V\left[{3w}^{\left(1\right)}+w^{\left(2\right)}\right]}=\frac{18v_{\varepsilon }^{\left(1\right)}v_{\varepsilon }^{\left(2\right)}-{2\left[v_{\varepsilon }^{\left(2\right)}\right]}^{2}}{3\left[3v_{\varepsilon }^{\left(1\right)}+v_{\varepsilon }^{\left(2\right)}\right]}$$ (10)

where $v_{\varepsilon }=\frac{w}{V}$ represents the strain energy density.

**FIGURE 2:**
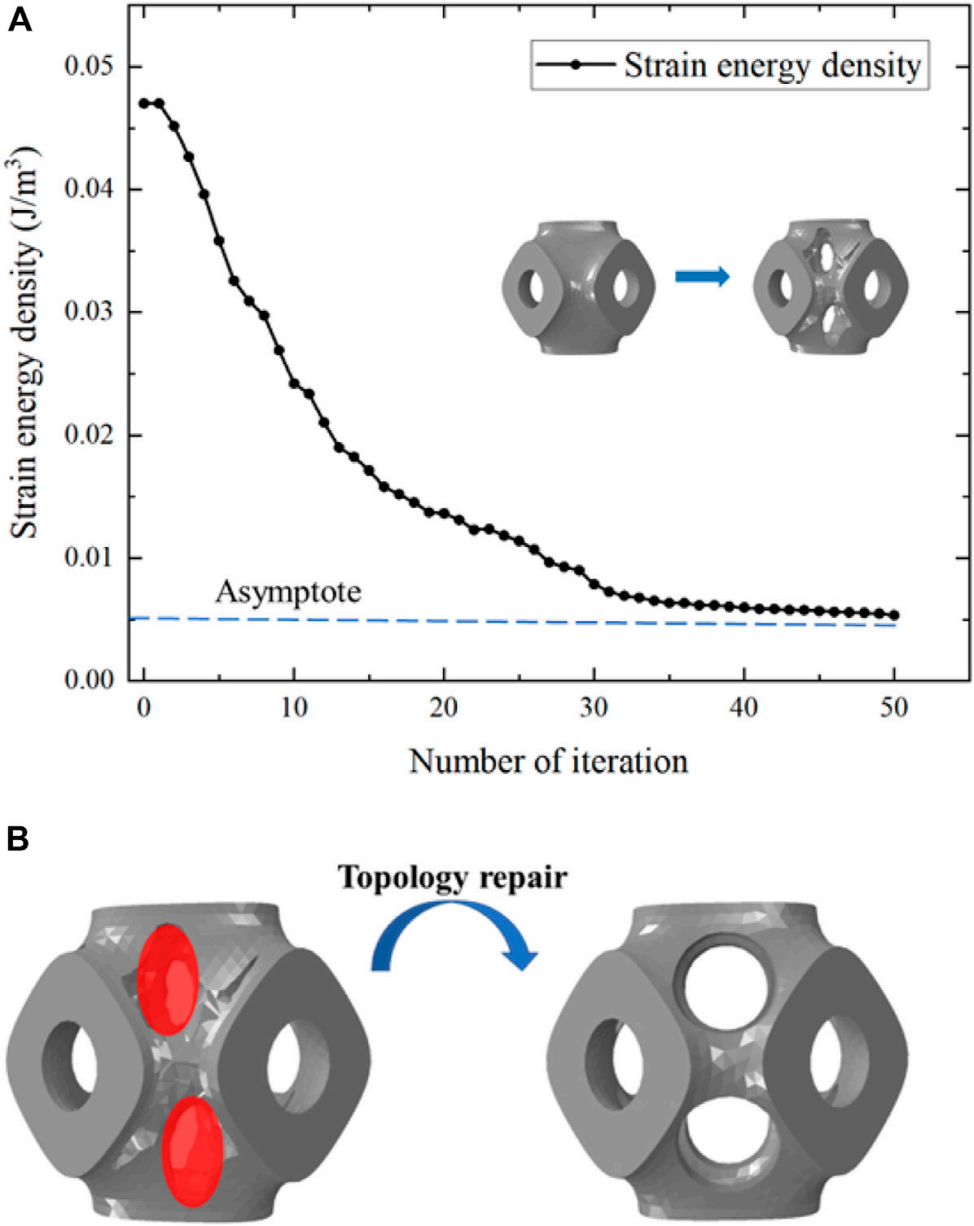
**(A)** Evolution of the strain energy density during the structural design. **(B)** Illustration of the geometric repair process.

To match the mechanical properties of cancellous bone, it is necessary to reduce the elastic modulus. Thus, the optimization objection is to find the minimum value of Eq. 10, which can be expressed as Eq. 11:

$$\left\{\begin{array}{l} \text{find }v_{\varepsilon }=\left(v_{\varepsilon }^{\left(1\right)},v_{\varepsilon }^{\left(2\right)}\right)\\ \min f\left(v_{\varepsilon }\right)=\frac{18v_{\varepsilon }^{\left(1\right)}v_{\varepsilon }^{\left(2\right)}-{2\left[v_{\varepsilon }^{\left(2\right)}\right]}^{2}}{3\left[3v_{\varepsilon }^{\left(1\right)}+v_{\varepsilon }^{\left(2\right)}\right]}\\ \text{subject to }0.5V_{0}\leq V\leq 0.8V_{0} \end{array}\right.$$ (11)

where V represents the volume of the Schwarz P scaffold after optimization, $V_{0}$ represent the original volume before optimization. Since the objection is to reduce the elastic modulus, the volume of the scaffold after optimization should be smaller than the original volume. On the other hand, in order to ensure that the optimized bone scaffold has enough volume to complete the function of mechanical support, the optimized volume should not be too small. Therefore, we set the constrain $0.5V_{0}\leq V\leq 0.8V_{0}$ . In the “Topology Optimization” part of ABAQUS (v2020, Dassault Systems SIMULIA Ltd., Providence, RI), we set the objection as to find the minimum value of Eq. 10; the constrain as $0.5V_{0}\leq V\leq 0.8V_{0}$ . It is worth noting that although the volume size is not directly related to the design variables, the volume constraint in the “Topology Optimization” part of ABAQUS is very important to achieve the optimization goal. Therefore, we summarize it into a unified optimization framework, which is Eq. 11. Moreover, the range of elastic modulus of human cancellous bone is 1.0–3.0 GPa. If the elastic modulus is lower than 1.0 GPa, the strength will not satisfy the requirement. Therefore, instead of directly calculating the minimum value of $f\left(v_{\varepsilon }\right)$ , we find that calculate the minimum values of $v_{\varepsilon }^{\left(1\right)}+v_{\varepsilon }^{\left(2\right)}$ can make sure the elastic modulus of the structure will not be lower than 1.0 GPa. The optimization process was carried out using ABAQUS with the following set: find the minimum value of $v_{\varepsilon }^{\left(1\right)}+v_{\varepsilon }^{\left(2\right)}$ when subject to $0.5V_{0}\leq V\leq 0.8V_{0}$ . The solid isotropic material with penalization (SIMP) was set. Figure 2A shows the calculation process of the optimization. To ensure that the obtained scaffolds can be fabricated using SLM, the thickness t should be limited within a certain range: t≥ 0.2. Materialise Magics (v24.0, Leuven, Belgium) is an AM-guided software, which can detect the thickness of the structure to ensure that it meets the manufacturing requirements. All the optimized structures in this paper are verified that the wall thickness meets the manufacturing requirements.

**FIGURE 3:**
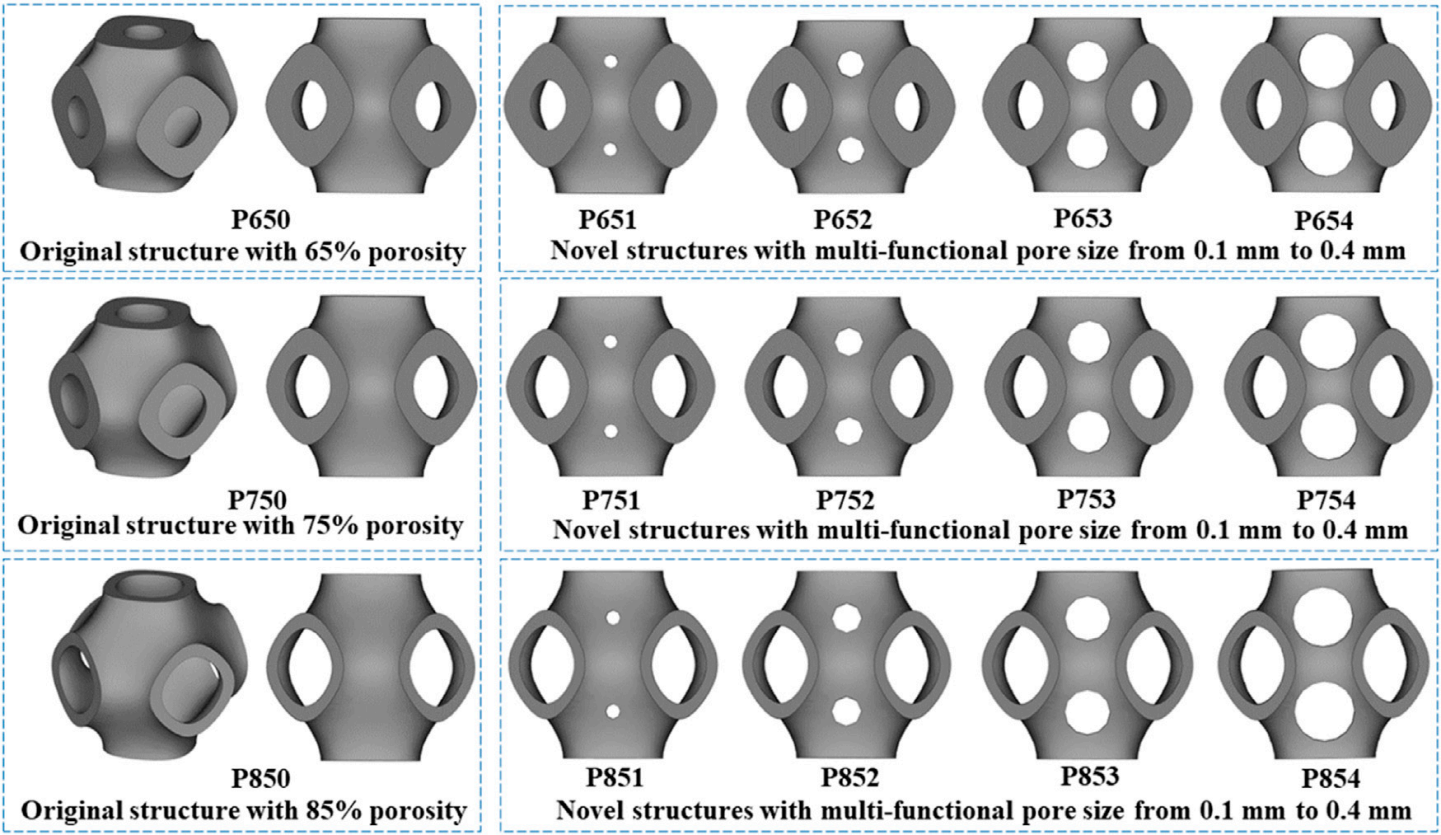
Three original scaffolds before optimization (named P650, P750, and P850) and the corresponding optimized novel scaffolds with multi-functional pore sizes from 0.1 mm to 0.4 mm (named P651–P654, P751–P754, and P851–P854).

The disadvantage of the SIMP method was that the boundary of the structure’s topology was not sufficiently clear and the structure was not able to be directly fabricated (Strömberg, 2021). Therefore, topology repair was necessary to ensure the scaffolds can be fabricated. This repair process involved adding multi-functional pores to the scaffolds where material had been removed, determined through ABAQUS calculations (Supplementary Figure 2.2B). To illustrate the structural design process, multi-functional pores with varying radii (0.1 mm, 0.2 mm, 0.3 mm, and 0.4 mm) were added (Figure 3). To maintain the cubic symmetry of the scaffold, eight multi-functional pores were added in each unit cell. P654 is taken as an example to illustrate the naming method for each scaffold: the letter “P” stands for the Schwarz P scaffold, the number “65” stands for the 65% porosity scaffold before the optimization, and “4” stands for the 0.4-mm radius of the multi-functional pores.

### 2.3 Additive manufacturing of Ti6Al4V Schwarz P structure

A group of Schwarz P structures with 65% porosity (P650–P654) were selected for manufacturing to carry out mechanical tests on them. Besides, in this process, it can also be proved that the designed new TPMS bone scaffold can be manufactured and has practical clinical significance. It is worth noting that all the TPMS bone scaffolds designed at 65% (P650-P654), 75% (P750-P754) and 85% (P850-P854) porosity meet the manufacturing requirements: t≥ 0.2 by verifications. The FE calculation results showed that the performance changes of each group of bone scaffolds are similar. Therefore, only a group of bone scaffolds with 65% porosity are selected to cross-verify with the results of FE calculation. The specimens were manufactured from Ti6Al4V powder using the SLM technique (Renishaw AM400, Wotton-under-Edge, United Kingdom). Due to the high melting point of Ti6Al4V materials, in the SLM process (Wauthle et al., 2015; Soro et al., 2019), the input laser power needed to be 280 W, and the scanning speed needed to be 7.3 mm/s. After the printing process, the samples were placed at a temperature of 1200°C for 2 hours, and the white corundum spraying process was carried out.

### 2.4 Mechanical simulations and analysis

To evaluate the mechanical behavior of the novel scaffolds, both FE simulations and mechanical experiments were performed. Based on the obtained elastic moduli, the Zener anisotropy indexed were calculated. The elastic moduli and Zener anisotropy indexes were used to evaluate the performance of the novel scaffolds.

For the FE simulations, the boundary condition of $\left\{\varepsilon^{\left(1\right)}\right\}$ was set as follows:

$$\left\{\begin{array}{l} {∆l}_{x}{|}_{x=l_{x}}=0.001l_{x}\\ {∆l}_{x}{|}_{x=0}={∆l}_{y}{|}_{y=0}={∆l}_{y}{|}_{y=l_{y}}={∆l}_{z}{|}_{z=0}={∆l}_{z}{|}_{z=l_{z}}=0 \end{array}\right.$$ (12)

The boundary condition of $\left\{\varepsilon^{\left(2\right)}\right\}$ was set as follows:

$$\left\{\begin{array}{l} {∆l}_{x}{|}_{z=l_{x}}=0.0005l_{z},{∆l}_{z}{|}_{x=l_{z}}=0.0005l_{x}\\ {∆l}_{z}{|}_{x=0}={∆l}_{y}{|}_{y=0}={∆l}_{y}{|}_{y=l_{y}}={∆l}_{z}{|}_{z=l_{z}}={∆l}_{x}{|}_{z=0}=0 \end{array}\right.$$ (13)

According to the derivation in Section 2.2, $C_{11}$ and $C_{12}$ could be obtained when the strain was set to $\left\{\varepsilon^{\left(1\right)}\right\}$ ; similarly, $C_{44}$ could be obtained when the strain was set to $\left\{\varepsilon^{\left(2\right)}\right\}$ . It is worth noting that the elastic constants should be averaged, which can be accomplished using Eq. 14:

$$C_{ij}=\bar{\sigma }=\frac{1}{V}\int_{V}\sigma_{ij}\text{dV}$$ (14)

where the sigma-bar of the stress $\bar{\sigma }$ represents the average of the stress values of all points in the entire volume region. Because in the process of FE calculation, the model is divided into many elements and nodes. In order to ensure the accuracy of the calculated stress values, the stress values of all nodes in the FE model are extracted, and the average value of these stress values is $\bar{\sigma }$ . The reliability of this data processing method has been confirmed by many previous studies (Feng et al., 2021; Peng et al., 2023).

**FIGURE 4:**
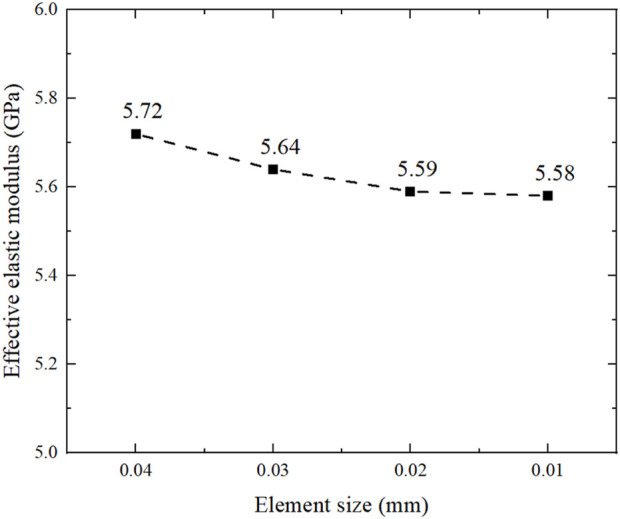
Convergence analysis of equivalent elastic modulus with different element sizes.

The scaffolds after topology repair were meshed using C3D8R elements with all element size of 0.01 mm. In order to ensure that the results based on this element size can be converged, the convergence analysis is shown in the Figure 4. The smaller the size of the element is, the greater number of meshes is, and the result tends to converge. Therefore, four different element sizes of 0.04 mm, 0.03 mm, 0.02 mm and 0.01 mm are taken to calculate the effective elastic modulus. According to the results obtained, the elastic modulus is 5.59 GPa when the element size is 0.02 mm and 5.58 GPa when the element size is 0.01 mm. Therefore, it can be considered that the result is convergent when the element size is 0.01 mm. At present, the material of bone scaffold is Ti6Al4V, which is widely used in clinic, that is, elastic modulus is 110.0 GPa and Poisson’s ratio is 0.3 (Montazerian et al., 2017). Then, the FE simulations were carried out in ABAQUS with an input elastic modulus of 110.0 GPa and Poisson’s ratio of 0.3. Eventually, the elastic moduli were calculated using Eq. 9.

The Zener anisotropy index, which is commonly used to evaluate the anisotropic properties of materials (Chen et al., 2019), is given by Eq. 15:

$$A=\frac{2C_{44}}{C_{11}-C_{12}}$$ (15)

After the FE analyses with the boundary conditions in Eq. 12 and Eq. 13, the stiffness matrix *C* was obtained, and then the Zener anisotropy index was calculated using Eq. 15. Next, the 3D anisotropic elastic responses of the Schwarz P scaffolds were visualized in MATLAB.

**FIGURE 5:**
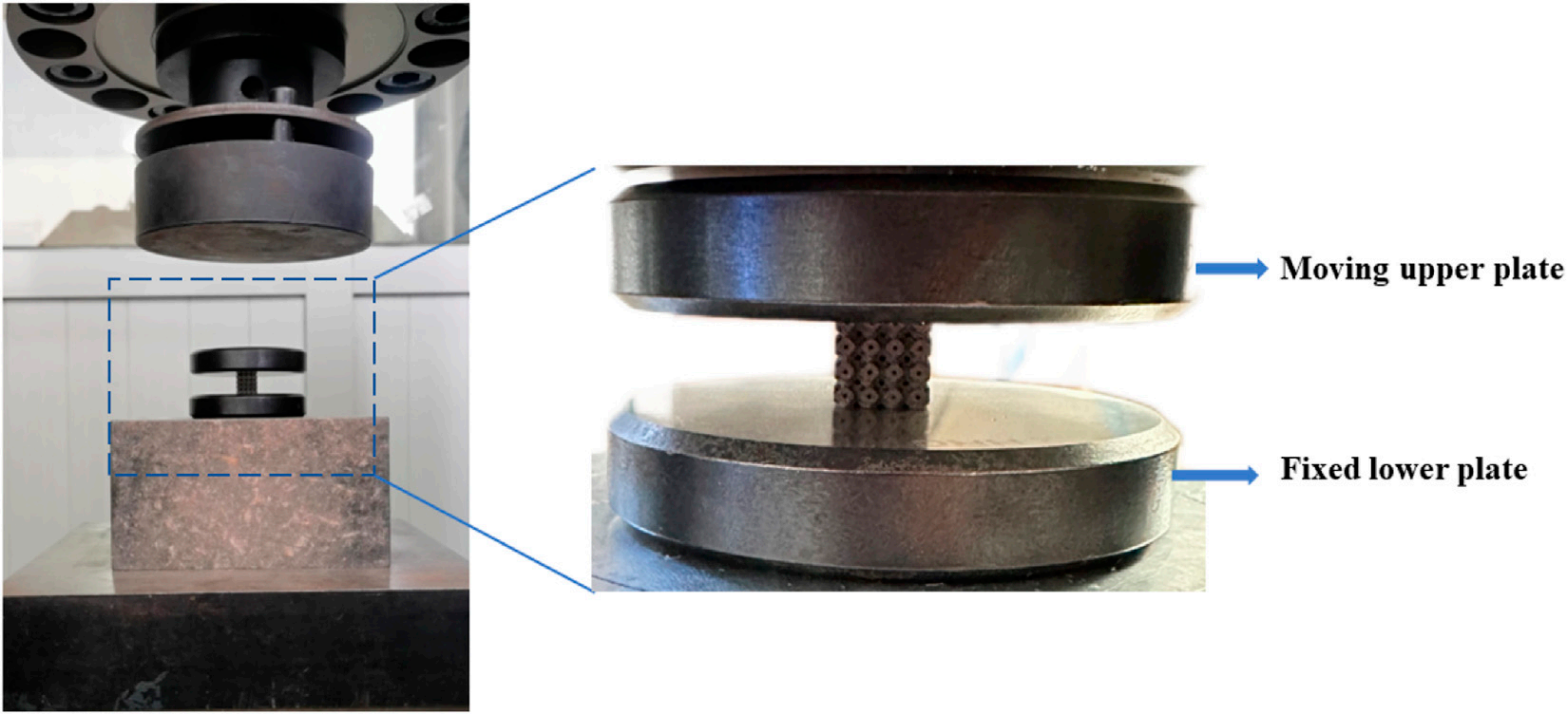
Experiment setup for the compression test: equipment and sample.

Quasi-static uniaxial compression tests were performed using INSTRON 5985 (Instron Company, Massachusetts, United States) and the loading speed was 0.5 mm/min according to the mechanical test standard ISO13314 (Ma et al., 2020). The samples were placed at the center of the lower fixture. The lower plate of the fixture remained fixed, while the upper plate was loaded at 0.5 mm/min, and the force and displacement were recorded using the sensors of the equipment (Figure 5). The stress was calculated by dividing the measured force by the cross-sectional area of the sample, and the strain was calculated by dividing the displacement by the height of the sample in the loading direction. The elastic modulus was calculated from the slope of the linear part of the stress–strain curve, and the yield stress was calculated using the 0.2% offset method. One camera was mounted in front of the samples to record the whole deformation process.

### 2.5 Mass transport simulations and analysis

Mass transport capacity (measured by permeability) is very important for bone scaffolds, because a high mass transport capacity is beneficial for the transmission in the scaffold, which is crucial for the growth of cells. Computational fluid dynamics (CFD) was used to simulate the process of transmission using COMSOL (v6.0, COMSOL Multiphysics, Stockholm, Sweden). The permeabilities of the novel scaffolds with multi-functional pores were calculated to evaluate the mass transport capacity.

**FIGURE 6:**
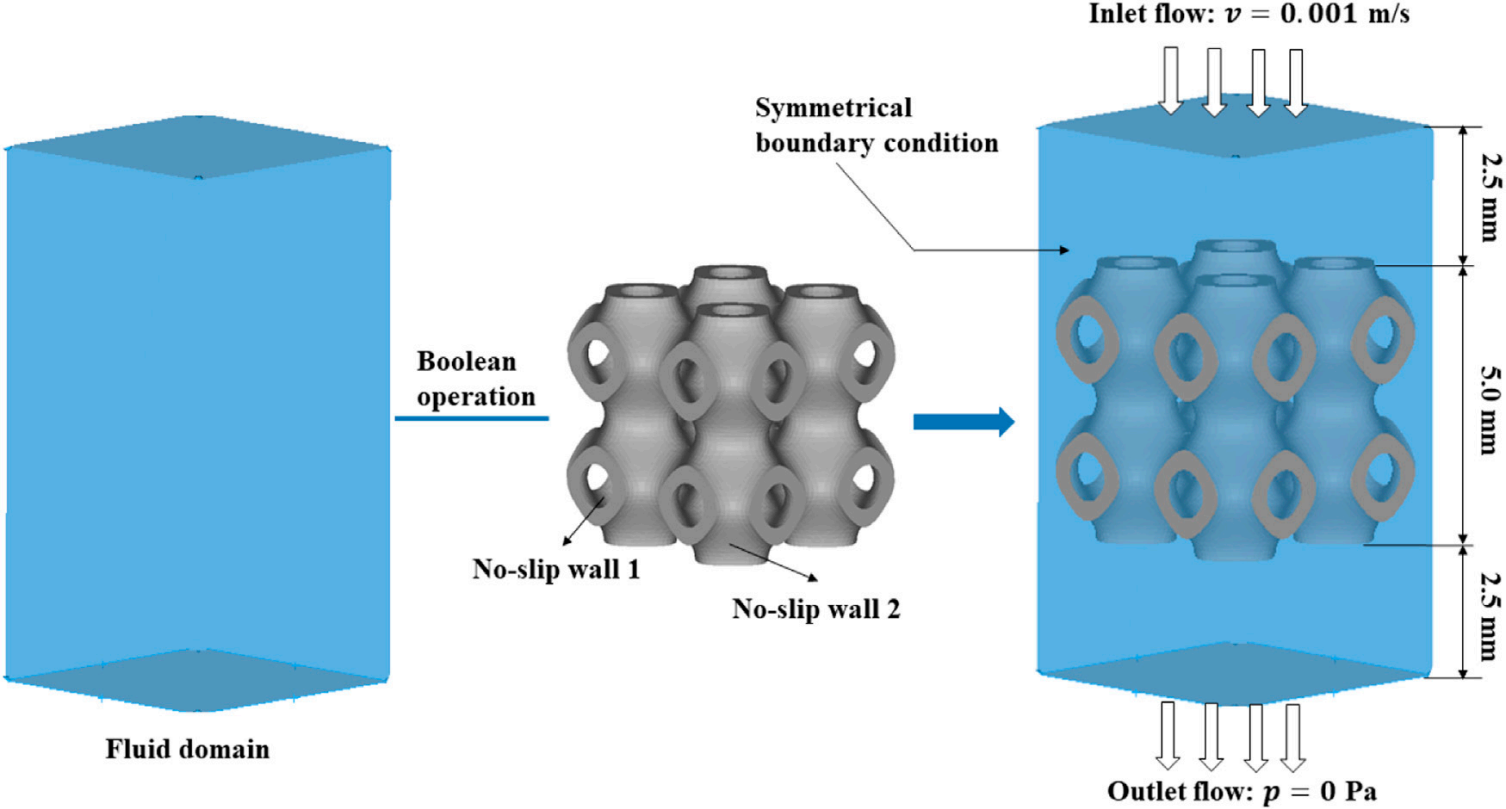
The modeling process of the fluid domain and the boundary conditions in CFD analysis.

All the structures were arrayed to 2 × 2 × 2 unit cells with a dimension of 5.0 mm × 5.0 mm × 5.0 mm. To avoid the boundary effect caused by the inlet and outlet area, a 5.0 mm × 5.0 mm × 2.5 mm virtual fluid domain was built at both the fluid flow inlet and outlet. Thus, a 5.0 mm × 5.0 mm × 10.0 mm parallelepipedal fluid domain was built (Figure 6). A common boundary condition for permeability was set with a flow rate of 0.001 m/s at the inlet and a pressure of 0.0 Pa at the outlet (Zhang et al., 2020; Qureshi et al., 2021). The external surface of the fluid domain and the surface of the Schwarz P scaffold were set as walls under no-slip state.

Darcy’s law is given by Eq. 16:

$$R_{e}=\frac{v\rho D}{\mu }$$ (16)

where $R_{e}$ is the Reynolds number; v is the velocity of the fluid (m/s); ρ is the density of the fluid (kg/m^3^); and D is the diameter of the pore (m).

The pressure drop between the inlet and outlet can be obtained from CFD calculation, and the permeability ( K ) of the structures can be calculated as:

$$K=\frac{v\mu L}{∆P}\;v=\frac{Q}{A}$$ (17)

where K is the permeability of the structure; v is the velocity of the fluid (m/s); μ is the dynamic viscosity coefficient of the fluid (Pa ∙ S); L is the length of the flow path (m); ∆P is the pressure drop (Pa); Q is the flow rate of scaffold (m^3^/s); and A is the cross-section area of the fluid domain (m^2^). The fluid properties of water were assumed to be ρ = 1000 kg/m^3^, μ = 0.001 Pa ∙ S, and v = 0.001 m/s.

## 3 Results

In this section, the geometric characteristics of the Schwarz P scaffolds are discussed first, including the difference in size between the designed and the SLM fabricated multi-functional pores. Then, the mechanical properties of all Schwarz P scaffolds under loading cases $\left\{\varepsilon^{\left(1\right)}\right\}$ and $\left\{\varepsilon^{\left(2\right)}\right\}$ are investigated using FE analysis in terms of elastic modulus and structural anisotropy. Additionally, the mass transport capacities of all Schwarz P scaffolds are investigated, including the pressure fields, velocity distributions, and structural permeabilities. Finally, the optimization results are presented, offering insights into how the scaffolds perform in comparison to natural cancellous bone and highlighting any improvements achieved through the design and optimization process.

### 3.1 Morphological characteristics

**FIGURE 7:**
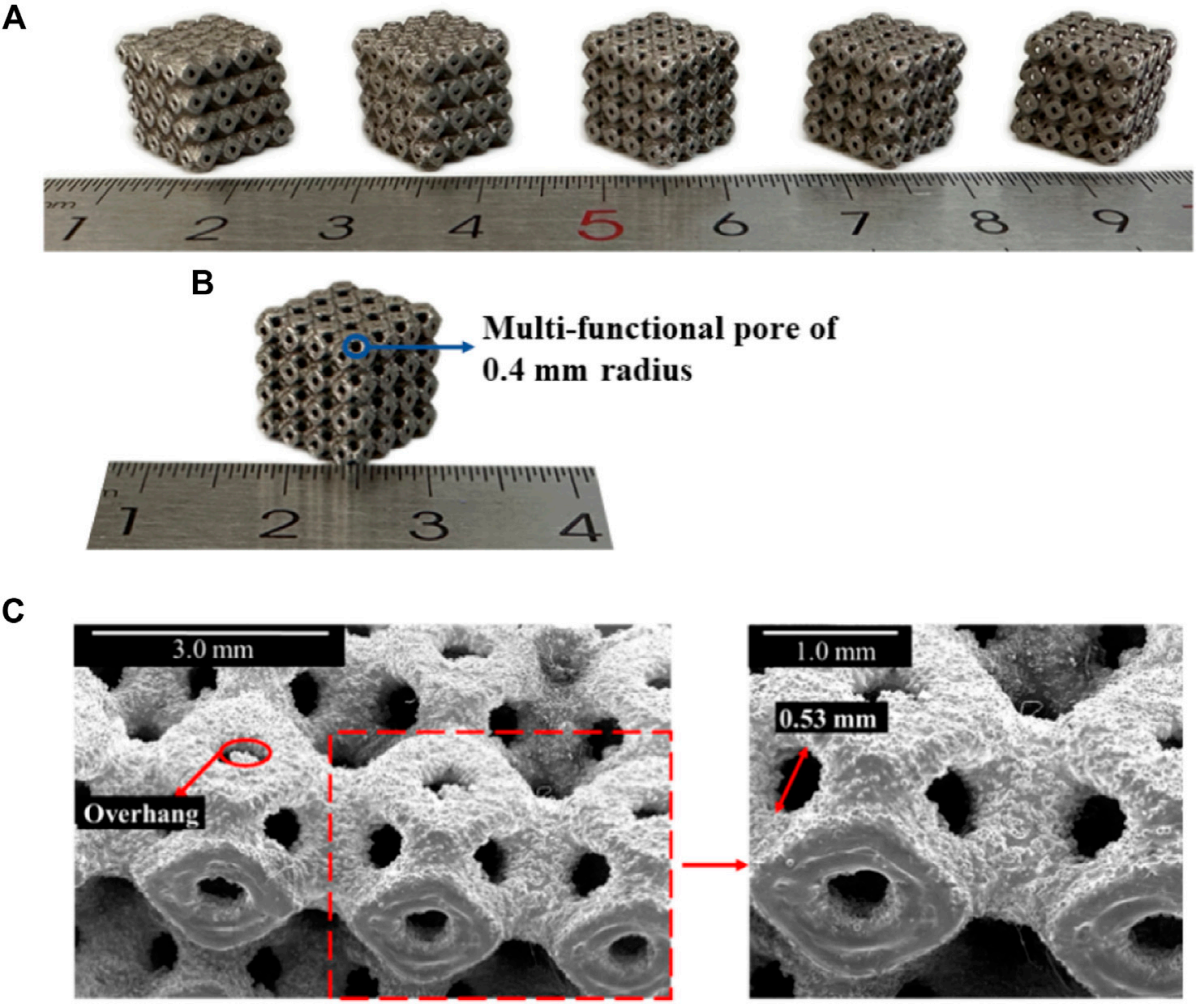
**(A)** SLM-fabricated Ti6Al4V specimens for P650–P654 scaffolds (P650 to P654 from left to right). **(B)** SLM-fabricated Ti6Al4V specimen for P654. **(C)** SEM image showing details of the fabricated sample of P654.

The SLM-fabricated structures are visualized in Figure 7A, and the sizes of the multi-functional pores are in the increasing order from the original structure to the novel optimized structure (P650–P654). To assess the accuracy of fabrication, the discrepancy between the fabricated specimens and the theoretical designs was examined, with particular focus on the P654 structure (Figure 7B) through a scanning electron microscope (SEM). The designed diameter of the multi-functional pore of P654 was 0.80 mm, and the theoretical observation size of the diameter was calculated to be 0.56 mm with the equation $R_{o}=R\times \cos$ 45 ° . The actual size of the diameter observed by SEM was 0.53 mm (Figure 7C), so the error between the fabricated and designed sizes was 5.4%. It is worth noting that the multi-functional pores were located on the curved surface, which could not be observed in a tangent plane direction. Therefore, the specimen was placed flat on the SEM observation platform, and the multi-functional pores were observed along a 45° angle.

### 3.2 Mechanical properties of the novel structures

**FIGURE 8:**
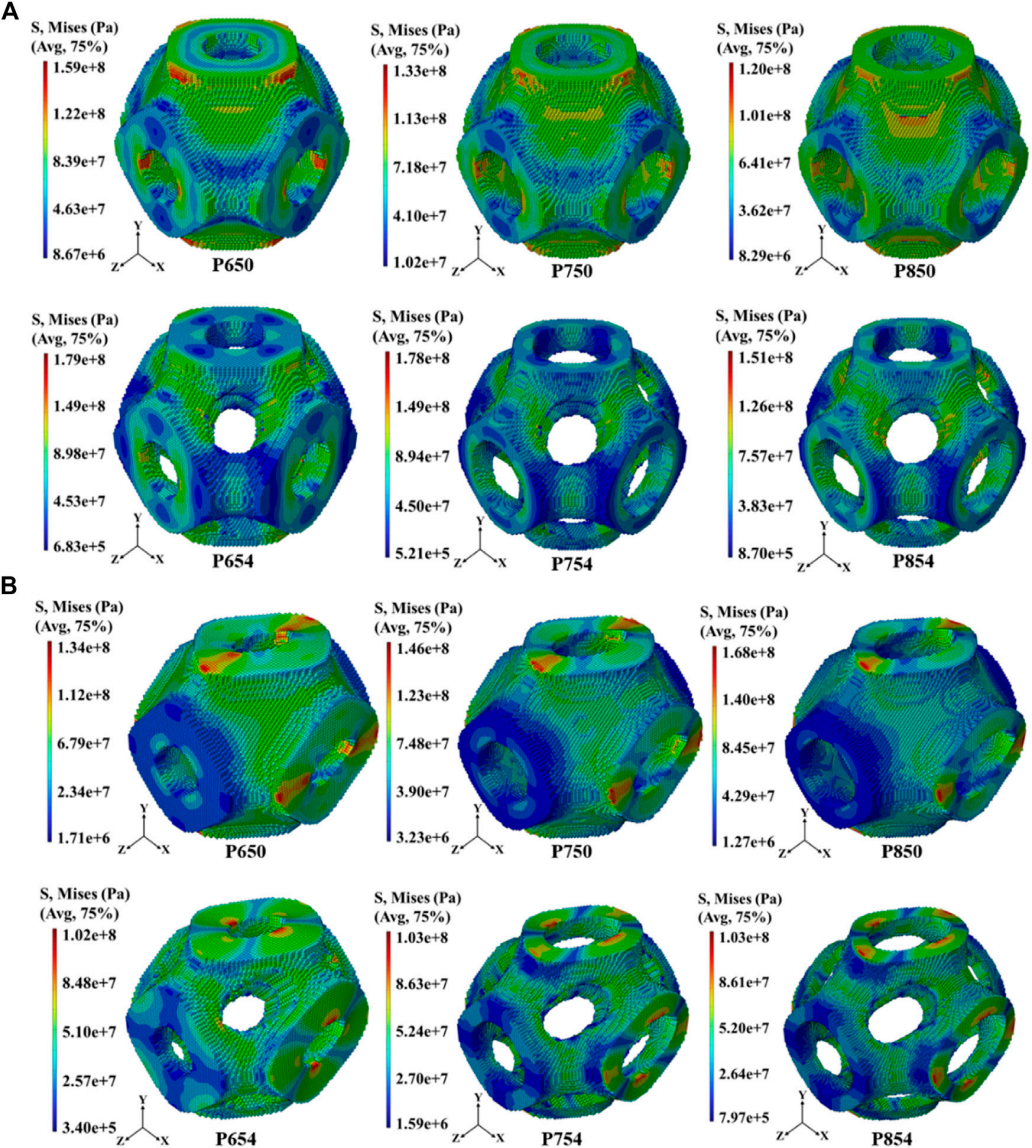
**(A)** Distribution of von Mises stress in the scaffolds before and after structural design at 65%, 75%, and 85% porosities under the uniaxial tensile load condition $\left\{\varepsilon^{\left(1\right)}\right\}$ . **(B)** Distribution of von Mises stress in the scaffolds before and after structural design at 65%, 75%, and 85% porosities under the shearing load condition $\left\{\varepsilon^{\left(2\right)}\right\}$ .

**FIGURE 9:**
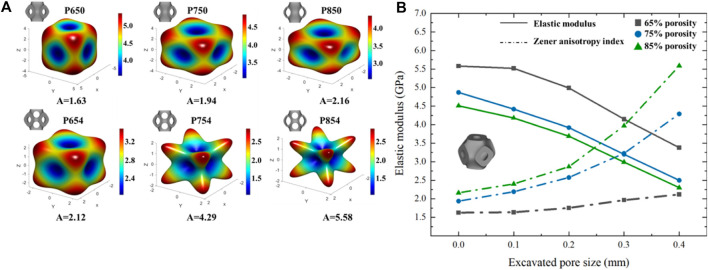
**(A)** Three-dimensional elastic modulus surface for different Schwarz P scaffolds. **(B)** Variation trend of structural elastic modulus and Zener anisotropy index with the increase of multi-functional pore size under different porosities.

The overall distribution of the von Mises stress under the tensile load condition $\left\{\varepsilon^{\left(1\right)}\right\}$ showed a decreasing trend after adding multi-functional pores to P654, P754, and P854. However, the overall distribution of von Mises stress under the shearing load condition $\left\{\varepsilon^{\left(2\right)}\right\}$ did not change much (Figure 8). With an increase in the radii of the multi-functional pores, the Zener anisotropy indexes of the novel scaffolds demonstrated a rising trend, while the elastic moduli displayed a decreasing trend. For instance, the Zener isotropy index of P650 was 1.63, and that of P654 was 2.12. The elastic moduli of P650, P750, and P850 were 5.58 GPa, 4.87 GPa, and 4.51 GPa, respectively, whereas the elastic moduli of P654, P754, and P854 were 3.38 GPa, 2.50 GPa, and 2.30 GPa, respectively. Notably, the elastic moduli of P754 and P854 fell within the range of 1.0–3.0 GPa, which is characteristic of human cancellous bone (refer to Figure 9B). To investigate the structural anisotropy, the effective stiffness was homogenized and every elastic modulus surface was colored according to the magnitude of the effective stiffness (Figure 9A).

**FIGURE 10:**
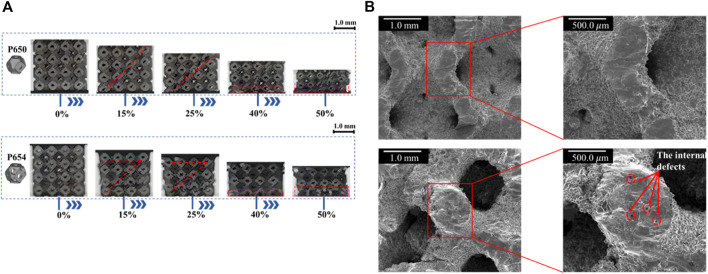
**(A)** Deformation behaviors of P650 and P654. **(B)** SEM views of the compressive fracture surfaces of P650 (upper) and P654 (lower).

The uniaxial compressive deformation behavior of the novel designed scaffold was basically consistent with that of the original scaffold. Both P650 and P654 scaffolds showed a shear band at the compressive strain of 0.15, which can be attributed to the slip. At the compressive strain of 0.40, the first fracture positions of the P650 and P654 structures were marked in the experimental results. Specifically, at this stage, the P654 structure exhibited a V-shaped shear band, while the P650 structure displayed a single diagonal shear band. Moreover, the fracture characteristics differed between the two structures. The fracture surface of the P650 specimen appeared smooth, whereas that of the P654 specimen was relatively rough, as depicted in Figure 10.

**FIGURE 11:**
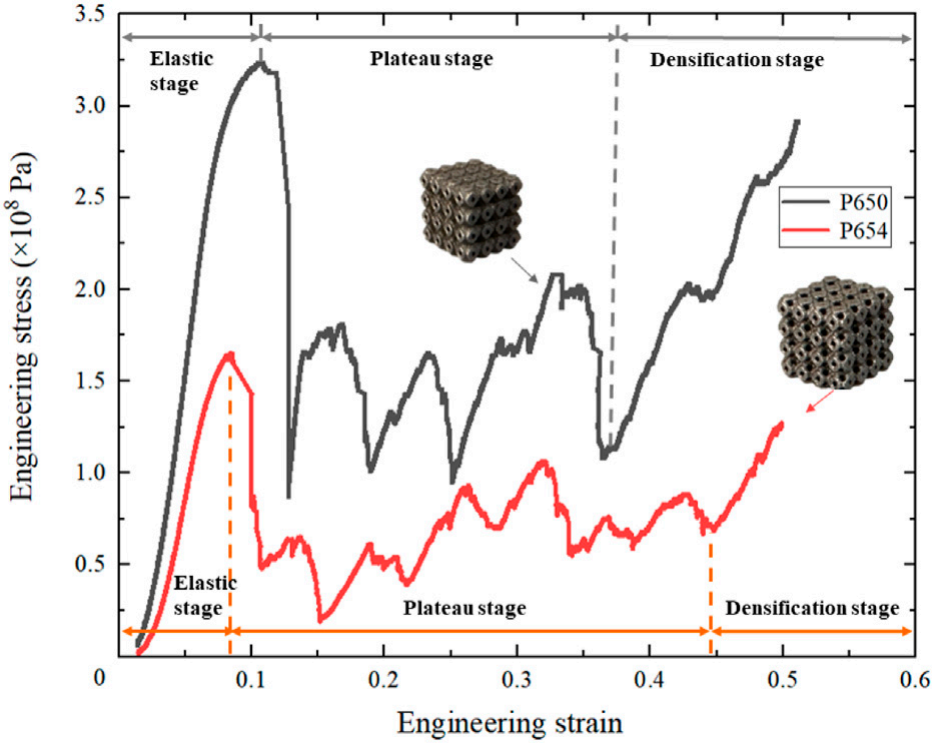
Stress–strain curves of the Schwarz P scaffolds: P650 and P654.

In Figure 11, the deformation behaviors of the P650 and P654 structures can be observed to follow a pattern consisting of three main stages: the elastic stage, plateau stage, and densification stage. Both curves started in the linear elastic stage, dropped sharply after reaching the peak stress, began a long plateau stage, and finally entered the densification stage from the strain of 0.4. In addition, the strains experienced by both structures at the ultimate compressive strength were found to be approximately the same.

**FIGURE 12:**
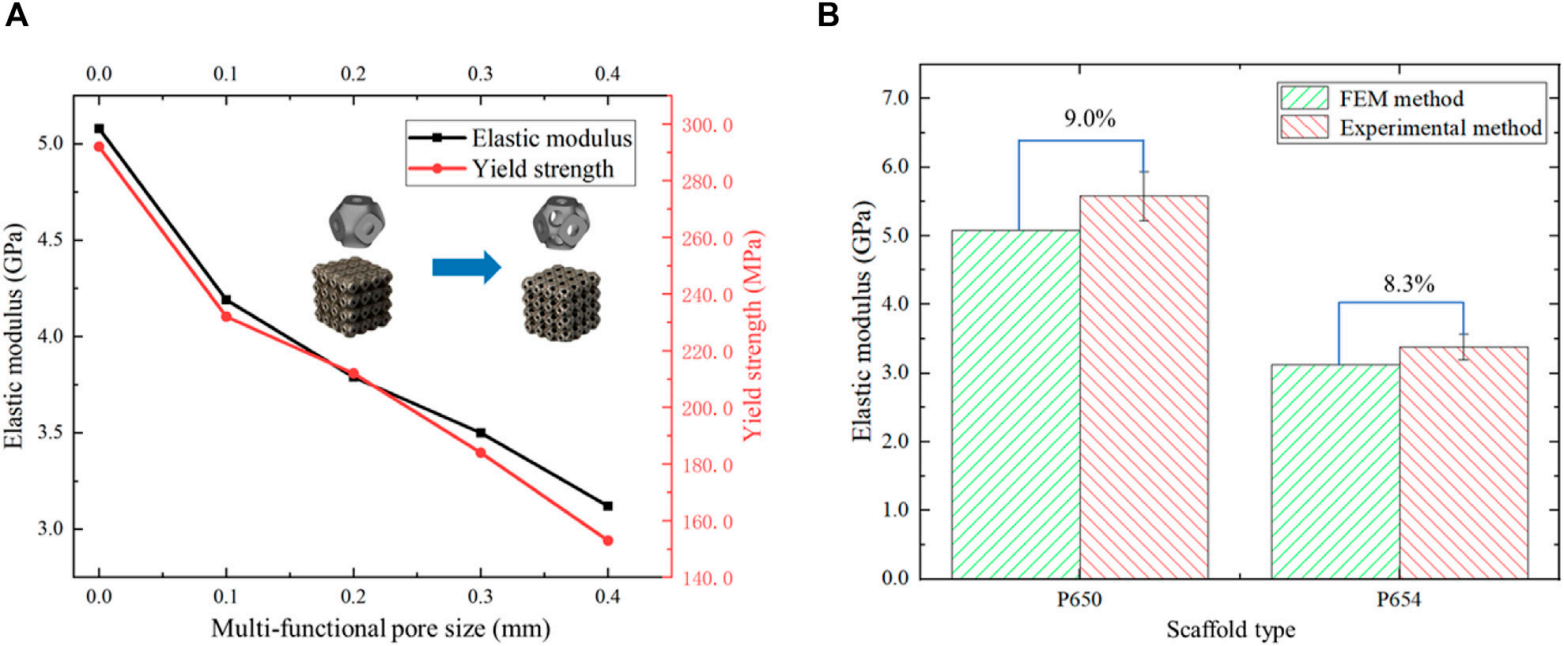
**(A)** Elastic moduli and yield strengths of scaffolds P650 to P654 calculated from experimental data. **(B)** Error in the elastic moduli of scaffolds P650 and P654 calculated by mechanical experiment and FE analysis.

The elastic modulus of the P650 scaffold was 5.58 GPa and that of the P654 structure was 3.38 GPa, showing the same trend as the FE simulation results. In addition, the yield strength of the P650 structure was 292.0 MPa, and that of the P654 structure was 153.0 MPa. The error between the measured elastic modulus and the FE simulation result was 9.0% for the P650 structure and 8.3% for the P654 structure (Figure 12).

### 3.3 Mass transport capacity of the novel new structures

**FIGURE 13:**
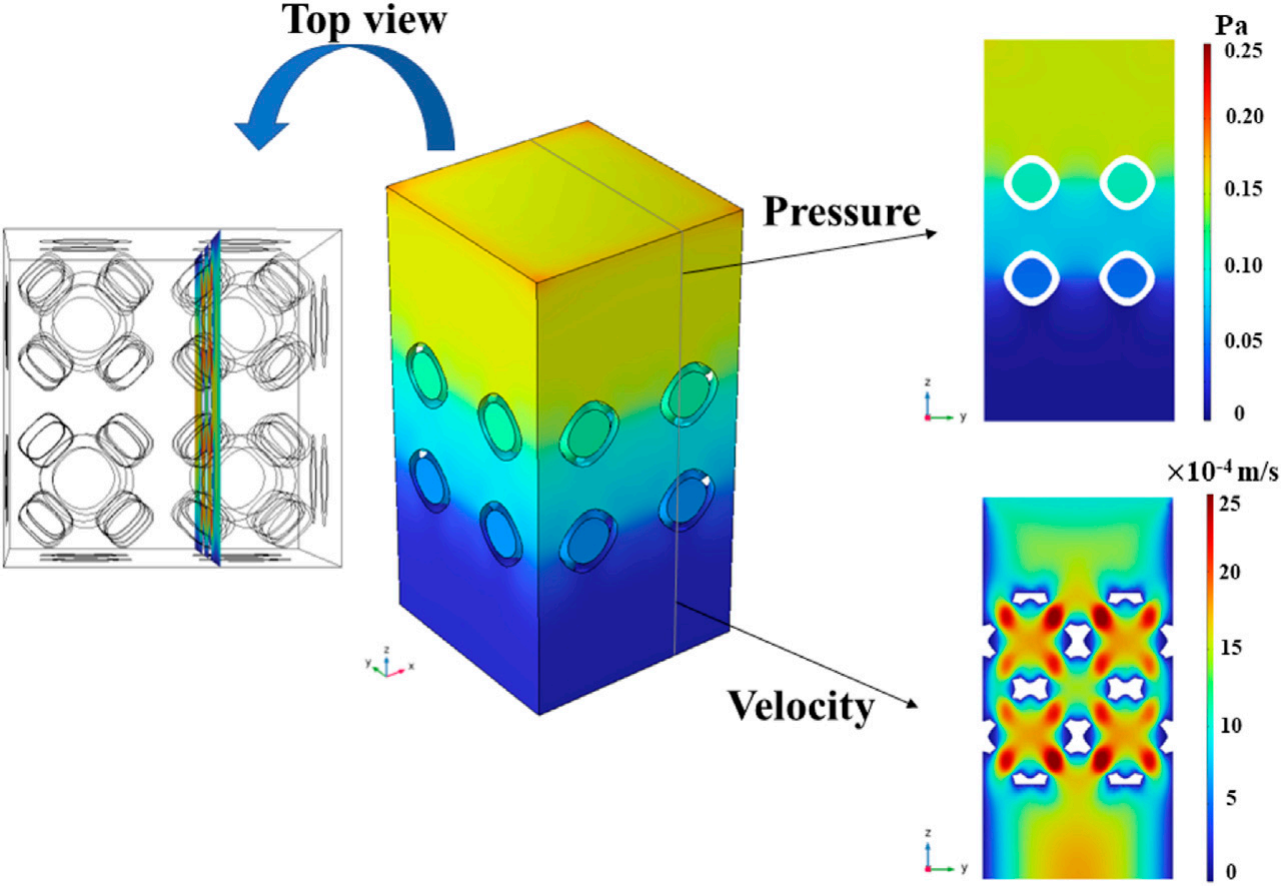
Pressure field and local flow velocity for the chosen cross section in the representative CFD model with symmetrical boundary conditions.

The pressure field in the entire model was homogenized along the vertical direction, indicating that the same pressure drop could be obtained by selecting any cross section. Since the multi-functional pores facilitated the fluid flow, the chosen cross-section position was strategically placed to intersect the centers of these pores, as illustrated in Figure 13.

**FIGURE 14:**
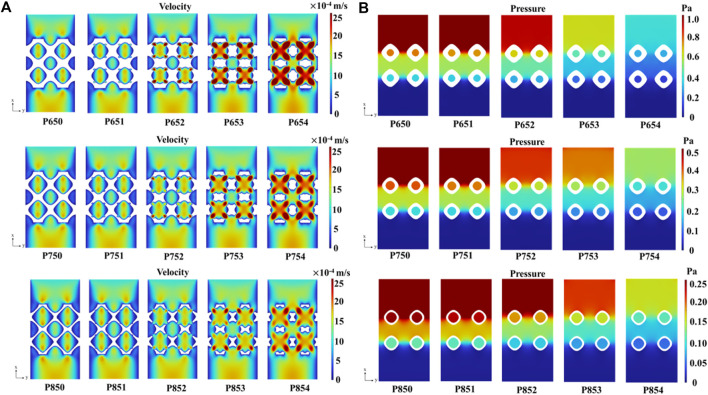
**(A)** Velocity field in the Schwarz P scaffolds with symmetrical boundary conditions. **(B)** Pressure distribution in the Schwarz P scaffolds with the symmetrical boundary conditions.

With an increase in the radius of the multi-functional pores, the fluid velocity experienced a significant rise, culminating in a maximum velocity 2.8 × 10^−3^ m/s in the P654 scaffold (Figure 14A). In addition, the pressure drop decreased with the increase of the radius of the multi-functional pore (Figure 14B). According to Darcy’s law shown in Eq. 17, the permeability of the scaffold was greatly improved by adding the multi-functional pores.

**FIGURE 15:**
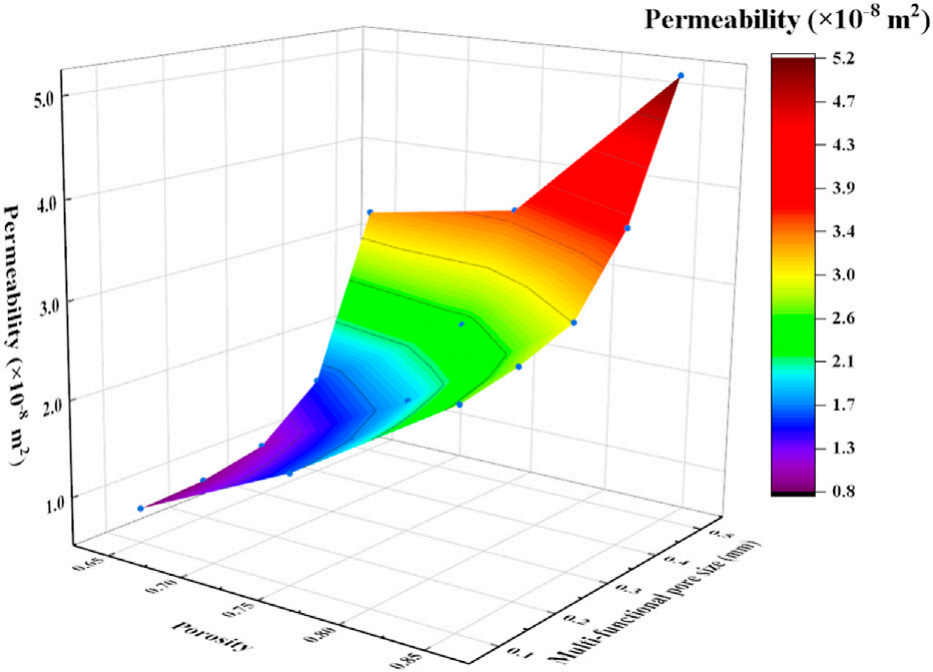
Effects of porosity and radius of multi-functional pores on the permeability of Schwarz P scaffolds.

The permeabilities of the scaffolds increased with the increase of porosity (Figure 15). For example, the permeability of the P650 structure was 8.58 × 10^–9^ m^2^, while that of the P850 structure was 2.65 × 10^–8^ m^2^. The permeability increased approximately three times with the increase of porosity from 65% to 85%. Furthermore, when holding porosity constant, the addition of multi-functional pores led to a significant enhancement in permeability. For example, the permeability of the P650 structure was 8.58 × 10^–9^ m^2^, and that of the P654 structure was 3.23 × 10^–8^ m^2^. The permeability increased approximately four times with the increase of the radius of the multi-functional pore from 0.1 mm to 0.4 mm.

**FIGURE 16:**
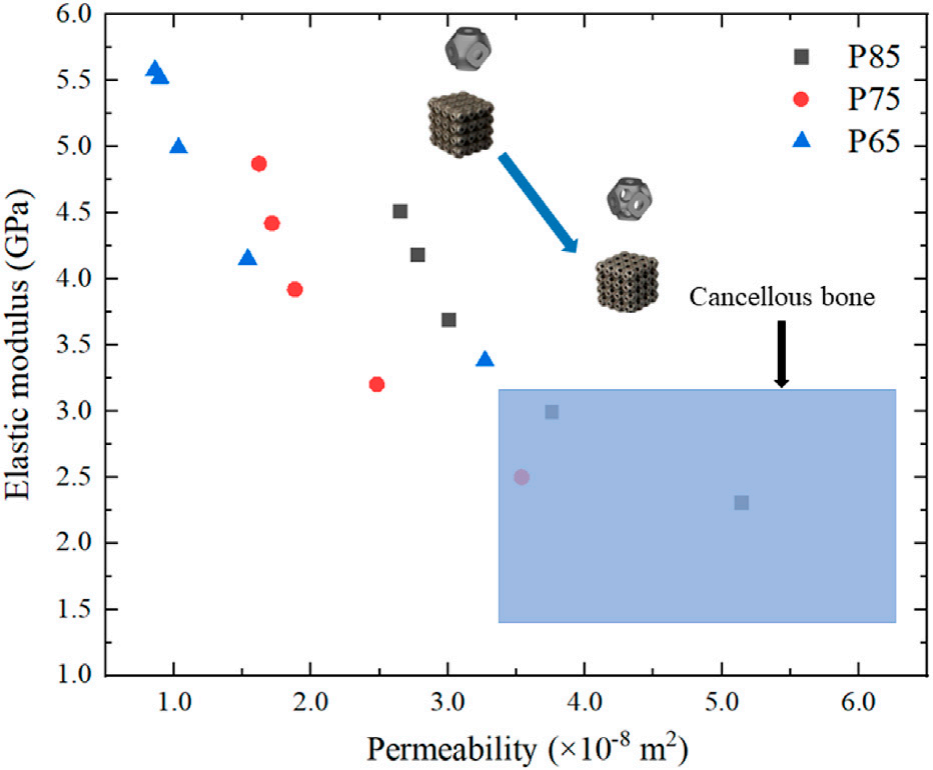
Optimization of the elastic modulus and permeability of Schwarz P scaffold and comparison with those of cancellous bone (the blue area is the range of the elastic modulus and permeability of cancellous bone).

Finally, the elastic moduli and permeabilities of the scaffolds were compared with those of cancellous bone (Figure 16). As a result of the design process, the coordinate points gradually shifted from the upper left corner (higher elastic modulus and lower permeability) of the graph to the lower right corner (lower elastic modulus and higher permeability). The elastic moduli and permeabilities of three structures, P754, P853, and P854, had been optimized to fall within the range observed for cancellous bone.

## 4 Discussion

Novel TPMS structures were designed and fabricated in this study. The morphological properties, mechanical properties, and mass transport capacities of the structures were investigated using FE simulations and mechanical experiments. In this section, the calculated results of the novel scaffolds are discussed and compared with those of cancellous bone, proving that the novel scaffolds in this study are ideal candidates for clinical applications.

### 4.1 Morphological characteristics

The error between the fabricated and designed values was 5.4%, indicating that the fabricated scaffolds were of good quality. However, it is necessary to discuss the possible reasons for the error. First, overhang structures were observed (Supplementary Figure 3.1C), which were the defects in the printing process generated by powder attachment. The side attachment was due to the partial melting of the powder around the contour line by laser tracking (Yan et al., 2012) and the bottom attachment was due to the improper thickness of the adjacent layers (Tian et al., 2017). Manufacturing defects such as overhang structures and residual powder are inevitable, and the same phenomena have been observed in other studies (Fan et al., 2021; Qiu et al., 2023). Second, the accuracy of the STL model and the internal structure of the specimen can also affect the manufacturing precision, but these factors are beyond the scope of this study. Although we can eliminate errors as much as possible, due to the limitations of AM technology, errors cannot be completely avoided. The error analysis of AM technology is another complex research field. Therefore, it may be possible to avoid errors by printing multiple times in the future, but in this study, considering the acceptable error of the multi-functional pore (5.4%), it is believed that the fabricated scaffolds meet the requirements of clinical applications.

### 4.2 Mechanical properties of the novel structures

The FE analysis results revealed that the novel scaffolds designed in this study do not produce stress concentration near the multi-functional pores, which will benefit the long-term service of the scaffolds (Supplementary Figure 3.2). Large stress will inevitably be generated near the multi-functional pores, but such a stress concentration phenomenon is not obvious, and it will not cause the structure to be damaged at the multi-functional pores. It is important to highlight that, in this study, mixed boundary conditions were employed instead of periodic boundary conditions to determine the scaffolds’ elastic moduli. This choice not only enhances computational efficiency but has also been validated for its reliable calculation accuracy in previous research (Feng et al., 2021; Baghous et al., 2023; Peng et al., 2023).

The Zener anisotropy indexes ( A ) of all scaffolds closely resembled those of cancellous bone, affirming the consistent anisotropic behaviors of the designed structures. The index A increased with the increase of the radius of the multi-functional pore and showed the following two trends. First, the larger the radius of the multi-functional pore, the faster the increase of index A (Supplementary Figure 3.3A). For structures with the same porosity of 85%, index A increased by 12.1% with the increase of the radius of the multi-functional pores from 0.1 mm to 0.2 mm. In contrast, the index A increased by 53.8% when the radius of the multi-functional pores increased from 0.2 mm to 0.3 mm. Second, the larger the porosity of the scaffold, the larger the difference between anisotropy indexes of the original and the novel designed scaffolds. For example, at 65% porosity, index A changed by 23% after novel design, whereas at 85% porosity, it changed by 61.4%. The index A of human cancellous bone is 1.0–4.0 (Kang et al., 2020). The index of the cancellous bone of the femur measured by CT scanning is 3.5 (Augat et al., 1998), and the index of the spine measured by the compressive testing method is 4.8 (Dendorfer et al., 2008). Thus, there is no unified conclusion on the range of the Zener anisotropy index of cancellous bone. However, the anisotropic behavior of P854 is still obvious, which may affect its mechanical properties. In conclusion, while the Zener anisotropy indexes of the novel scaffolds align with cancellous bone standards, the challenge of managing anisotropy during structural design necessitates further exploration and resolution.

The deformation behavior of the newly designed structure closely mirrored that of the original structure, as illustrated in Supplementary Figure 3.4. Within the strain range of 0–0.1, both structures exhibited stress-strain relationships that were predominantly linear. Notably, at a strain of 0.1, the stresses of P650 and P654 experienced significant drops to 27.4% and 51.3% of their peak stress levels, respectively. This phenomenon was related to the brittle failure of pillars in the Ti6Al4V lattice treated by SLM (Kadkhodapour et al., 2017), and this same phenomenon has been observed in previous studies. For example, Qiu et al. (2023) reported that the stress dropped to 39.4% of the peak stress, and Rezapourian et al. (Rezapourian et al., 2023) reported that the stress dropped to 8.9% of the peak stress. As the stress decreased, both the P650 and P654 structures exhibited a shear band along a diagonal direction of 45°, and P654 produced an additional horizontal shear band (Supplementary Figure 3.4A). The shear band along the diagonal was caused by the diagonal distribution of the maximum local curvature of the Schwarz P structure (Fan et al., 2021). In the plateau stage, the stresses were lower than the ultimate strength and increased in waves. Subsequently, the two structures entered the densification stage, and the stress–strain curve showed an upward trend and reached its initial peak. As the strain increased, the fracture zone of the structure continued to expand, resulting in a state of collapse in the lower layer and a state of yield in the upper layer. The P650 structure displayed smooth and flat fracture characteristics, indicating that the structure’s fracture type was primarily due to tensile deformation (Supplementary Figure 3.4B). Furthermore, some internal defects were observed on the fracture cross section of the P654 structure, which may have led to the formation of the horizontal shear band of P654. Similar internal defects have been documented in other studies as well, indicating a potential influence on the mechanical behavior and failure mode of the structure (Fan et al., 2021; Qiu et al., 2023).

The compressive strength and yield strength of the novel scaffolds were compared with those of cancellous bone, indicating that the novel scaffolds had sufficient strength. The compressive strength of cancellous bone is 5.8 MPa and the yield strength of cancellous bone is 4.1 MPa (Syahrom et al., 2011). The P654 structure had compressive strength of 165.0 MPa and yield strength of 153.0 MPa. Therefore, the novel scaffolds met the strength requirements. The error between the FE method and experimental method was 9.0% for the P650 structure and 8.3% for the P654 structure. The error between the two methods evaluated by Jia et al. (2020) fluctuated between 8.0% and 20.0%, so the error in this study was within the acceptable range.

### 4.3 Mass transport capacity of the novel structures

With the increase of the radius of the multi-functional pore, the velocity of the fluid in the scaffold increased gradually (Supplementary Figure 3.8A). When the radius of the multi-functional pore reached 0.4 mm, the internal structure was completely connected, which significantly improved the permeability of the scaffold. In the past, the method to improve permeability was to increase porosity, but the possible porosity for a certain TPMS scaffold (Maskery et al., 2018) is limited to a certain range. For example, the permeability of P650 was 8.58 × 10^–9^ m^2^, and the permeability of P850 was 2.65 × 10^–8^ m^2^. By increasing the porosity from 65% to 85%, the permeability of the structure was increased approximately 3.1 times. However, the permeability of P654 was 3.27 × 10^–8^ m^2^. Thus, the permeability of the P650 scaffold increased 3.8 times after adding multi-functional pores with a 0.4-mm radius to the scaffold. Therefore, the novel designed scaffolds proposed in this study introduce a new way to control the permeability of TPMS scaffolds.

After adding the multi-functional pores to the scaffolds, the elastic moduli and permeabilities of P754, P853, and P854 fall within the corresponding ranges of cancellous bone (Supplementary Figure 3.10). In addition, with the progress of adding multi-functional pores, the change of permeability was much larger than the change of elastic modulus. From the P650 scaffold to the P654 scaffold, the elastic modulus decreased by 39.0% and the permeability increased by 281.0%. Previous studies have shown that the permeability of a TPMS scaffold is more sensitive to pore size than is the elastic modulus (Sutradhar et al., 2016). The results shown in Supplementary Figure 3.10 also prove that the design method proposed in this study can expand the design domain of TPMS structure. To be specific, since the TPMS structure is controlled by mathematical equation such as Eq. 1, it means that the variable parameters in Eq. 1 is the only variable, which leads to great limitations in the design domain of TPMS structures. To solve this problem, novel TPMS structures such as functional graded TPMS structures are designed in previous studies to broaden the design domain (Fan et al., 2021; Liu et al., 2022). The optimal design method proposed in this study introduced a new variable: multi-functional pore, it can broaden the design domain of TPMS structure, so more different TPMS bone scaffolds can be designed. As shown in Supplementary Figure 3.10, the bone scaffolds that possess elastic modulus and permeability meet the requirements of cancellous bone can be designed by this method, which are new scaffolds cannot be obtained in the previous design domain.

Although promising results were obtained in this study, there are still some limitations that should be resolved in the future. First, the novel design method proposed in this study is only suitable for symmetric structures such as TPMS structures. Because the TPMS structures are symmetrical, its stiffness matrix C can be simplified and the optimization framework was based on the simplified stiffness matrix C . Second, only the Schwarz P scaffolds were evaluated in this study, and other types of TPMS scaffolds remain to be analyzed. Last but not least, at present, only a group of bone scaffolds with 65% porosity have been manufactured and mechanical experiments have been carried out. The manufacture of bone scaffolds with more porosity needs to be carried out in the future.

## 5 Conclusion

To address the problems of high elastic modulus and low permeability in current bone scaffolds, novel TPMS bone scaffolds with optimization-guided multi-functional pores were designed in this study. The performances of the novel TPMS scaffolds were investigated using experimental characterization and numerical simulations. The main conclusions are as follows:

(1) The effective elastic modulus reduced from 5.58 GPa (original scaffold) to 3.38 GPa (novel designed scaffold), resulting in lower stress shielding.
(2) The multi-functional pores greatly improved the mass transport capacities of the TPMS scaffolds by providing new pores on the walls. The permeability increased from 8.58 × 10^−9^ m^2^ (original scaffold) to 5.14 × 10^−8^ m^2^ (novel designed scaffold)
(3) The deformation mode of the novel TPMS bone scaffolds at 65% porosity remained unchanged, which ensured that the new scaffolds can be used as stably as the previous scaffolds. The compressive strength and yield strength of the structure met the clinical requirements, i.e., the scaffolds need to have enough strengths to ensure that they will not break easily.
(4) The novel scaffolds expanded the design domain of TPMS-based bone scaffolds, providing a promising new method for the design of high-performance bone scaffolds.

## Data Availability

The original contributions presented in the study are included in the article/Supplementary Material, further inquiries can be directed to the corresponding author.
