# Supplementary figures and images for: Design of novel triply periodic minimal surface (TPMS) bone scaffold with multi-functional pores: lower stress shielding and higher mass transport capacity
Source: Front Bioeng Biotechnol. 2024 Jun 26;12:1401899. doi: 10.3389/fbioe.2024.1401899 (PMC11238189; doi:10.3389/fbioe.2024.1401899)

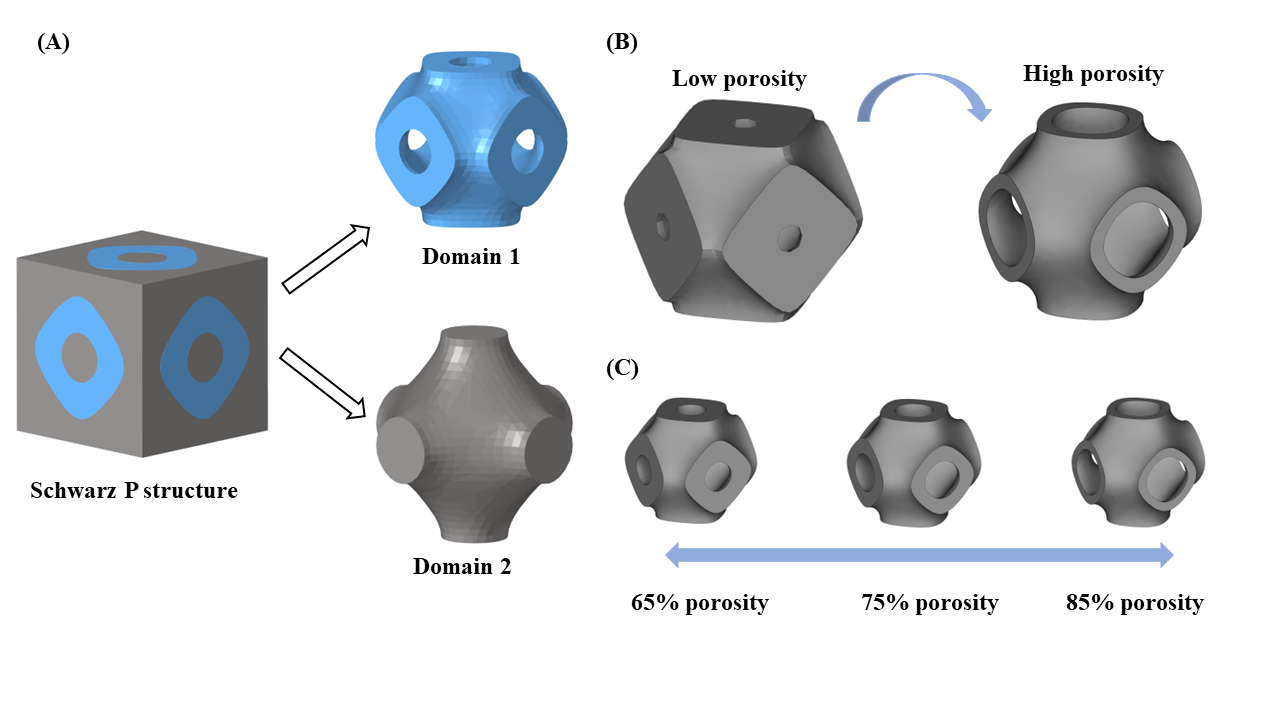

Supplement: Supplementary file 1 [file DataSheet1.ZIP › Supplementary Material Presentation/Figure 2.1.png]

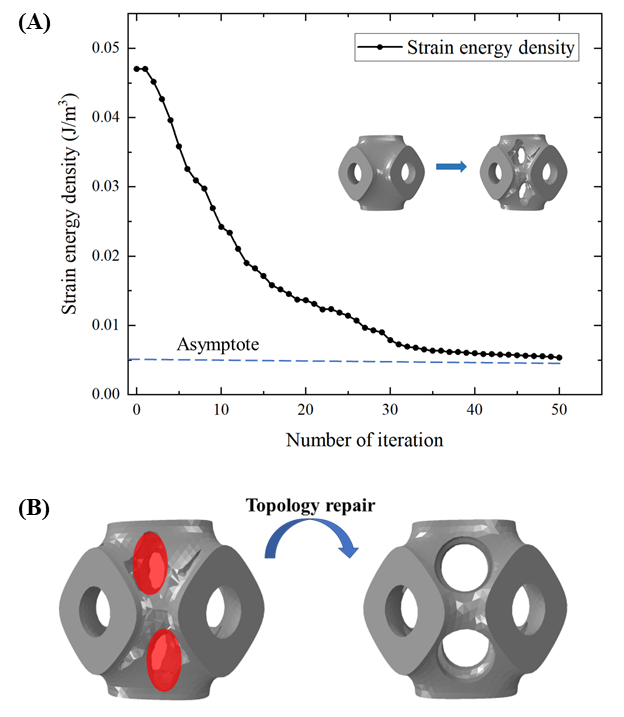

Supplement: Supplementary file 1 [file DataSheet1.ZIP › Supplementary Material Presentation/Figure 2.2.png]

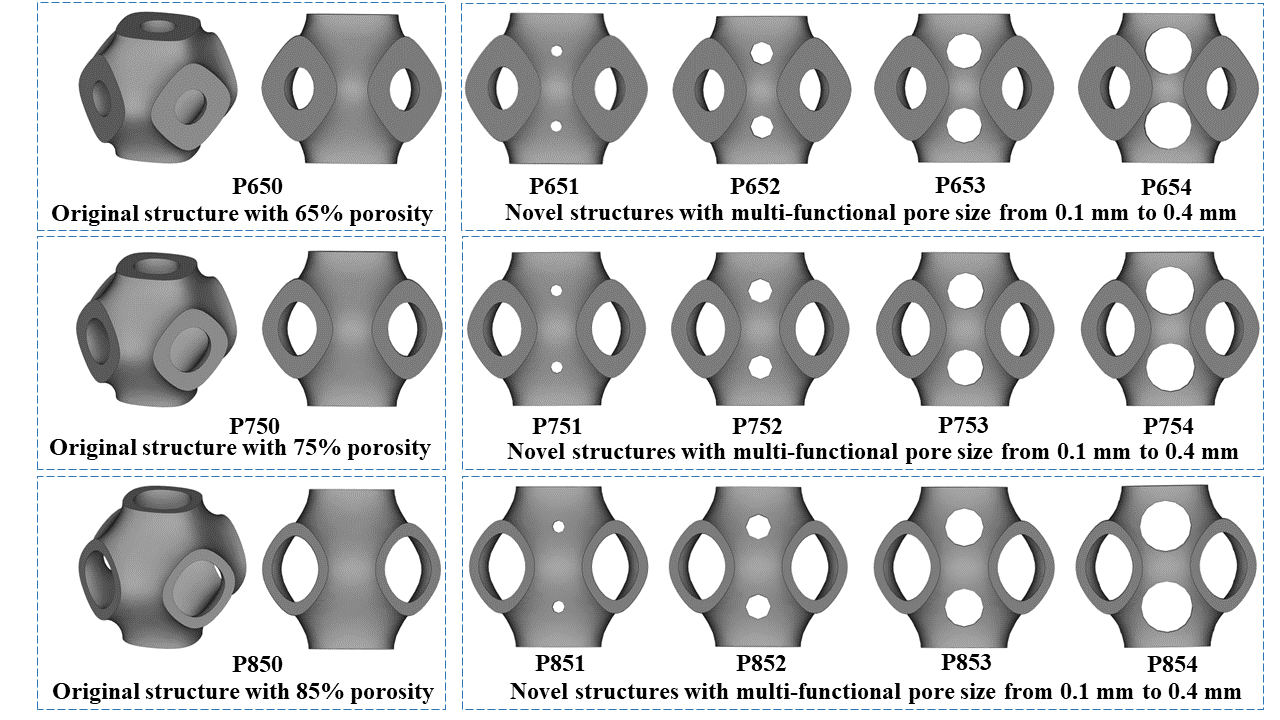

Supplement: Supplementary file 1 [file DataSheet1.ZIP › Supplementary Material Presentation/Figure 2.3.png]

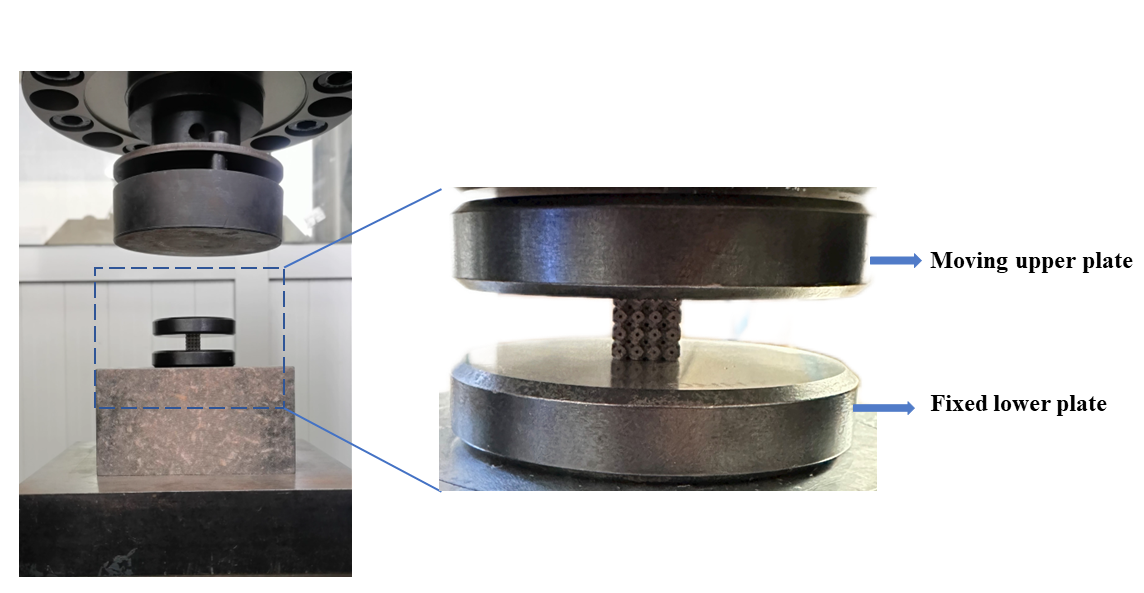

Supplement: Supplementary file 1 [file DataSheet1.ZIP › Supplementary Material Presentation/Figure 2.4.png]

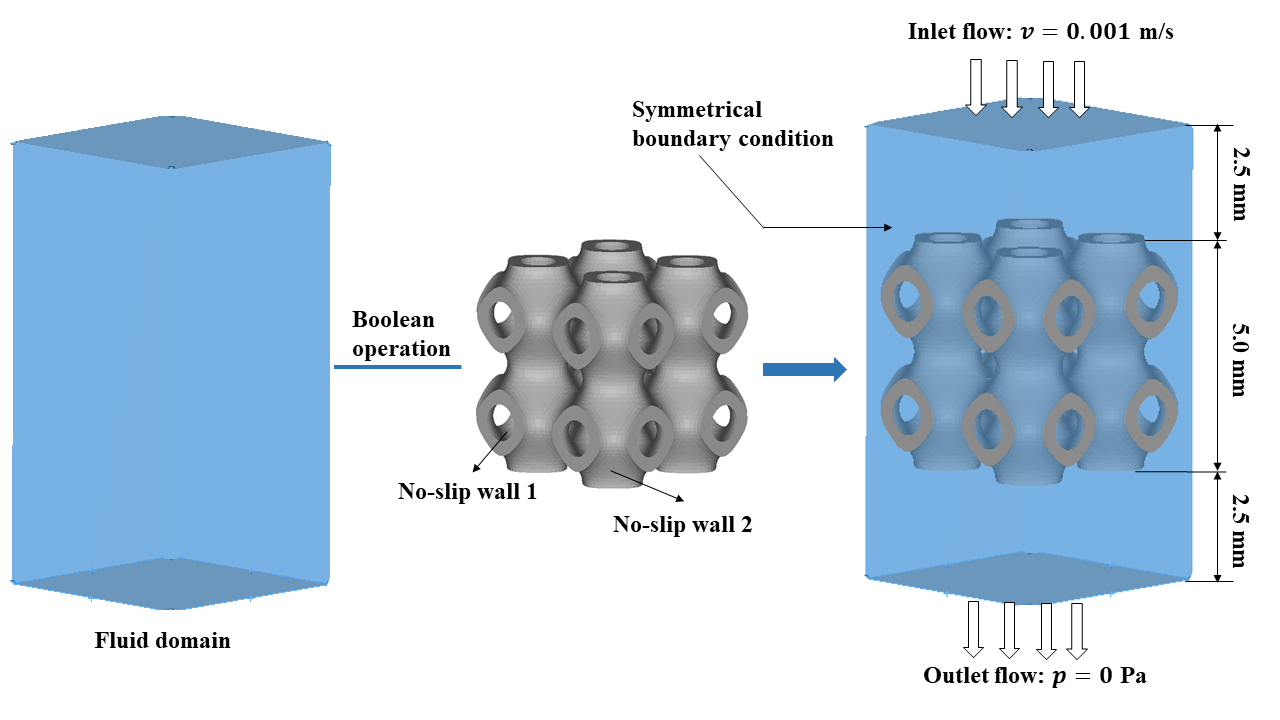

Supplement: Supplementary file 1 [file DataSheet1.ZIP › Supplementary Material Presentation/Figure 2.5.png]

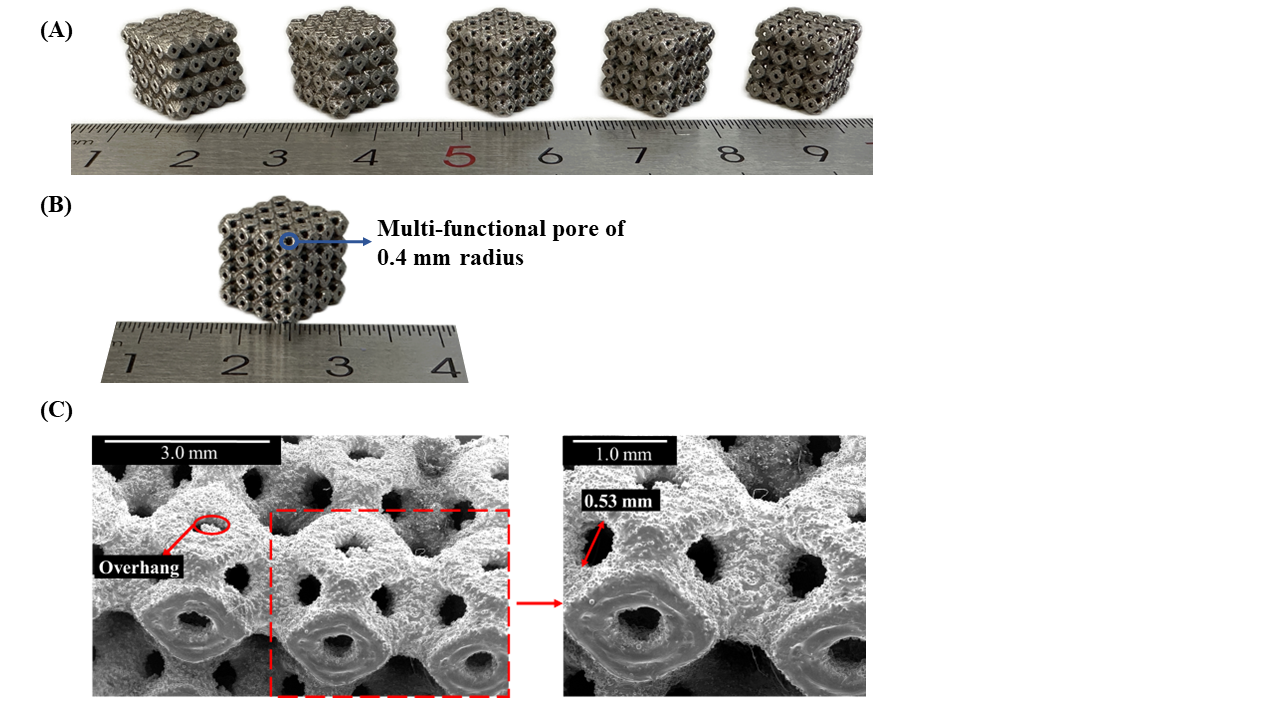

Supplement: Supplementary file 1 [file DataSheet1.ZIP › Supplementary Material Presentation/Figure 3.1.png]

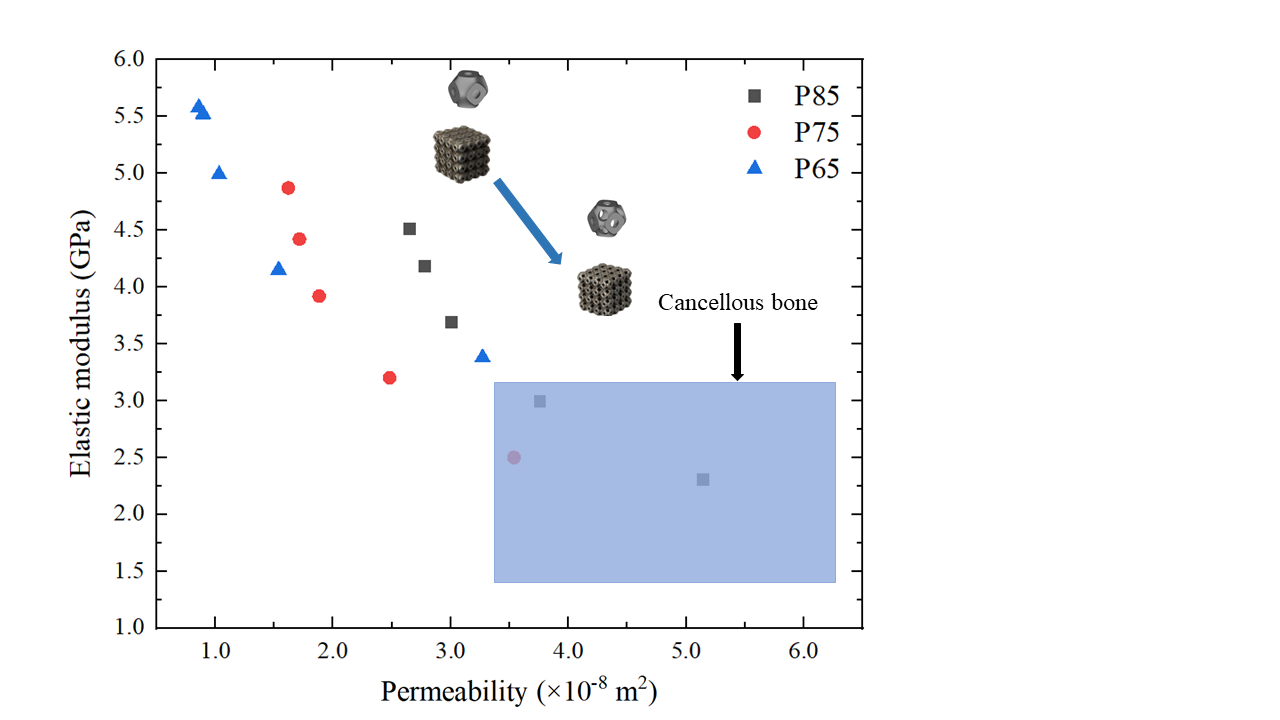

Supplement: Supplementary file 1 [file DataSheet1.ZIP › Supplementary Material Presentation/Figure 3.10.png]

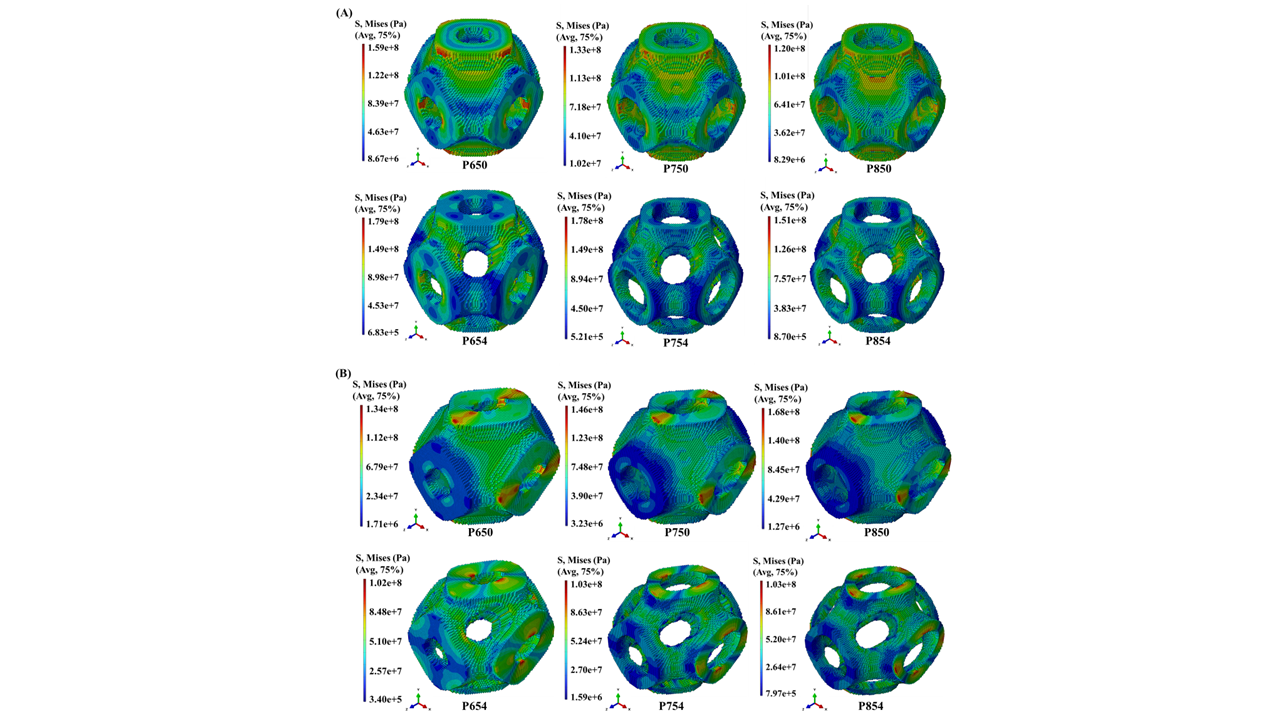

Supplement: Supplementary file 1 [file DataSheet1.ZIP › Supplementary Material Presentation/Figure 3.2.png]

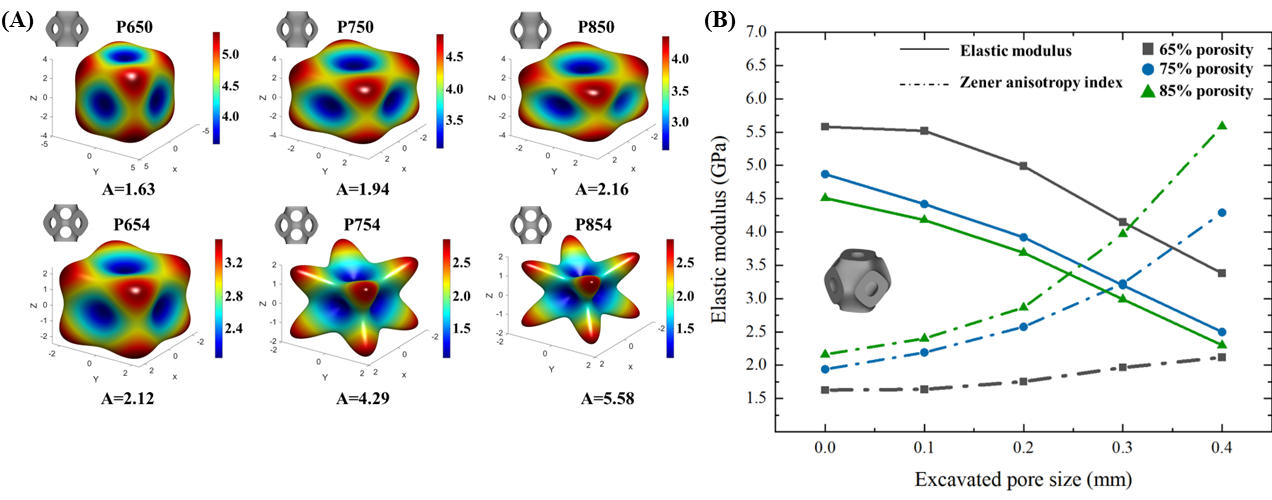

Supplement: Supplementary file 1 [file DataSheet1.ZIP › Supplementary Material Presentation/Figure 3.3.png]

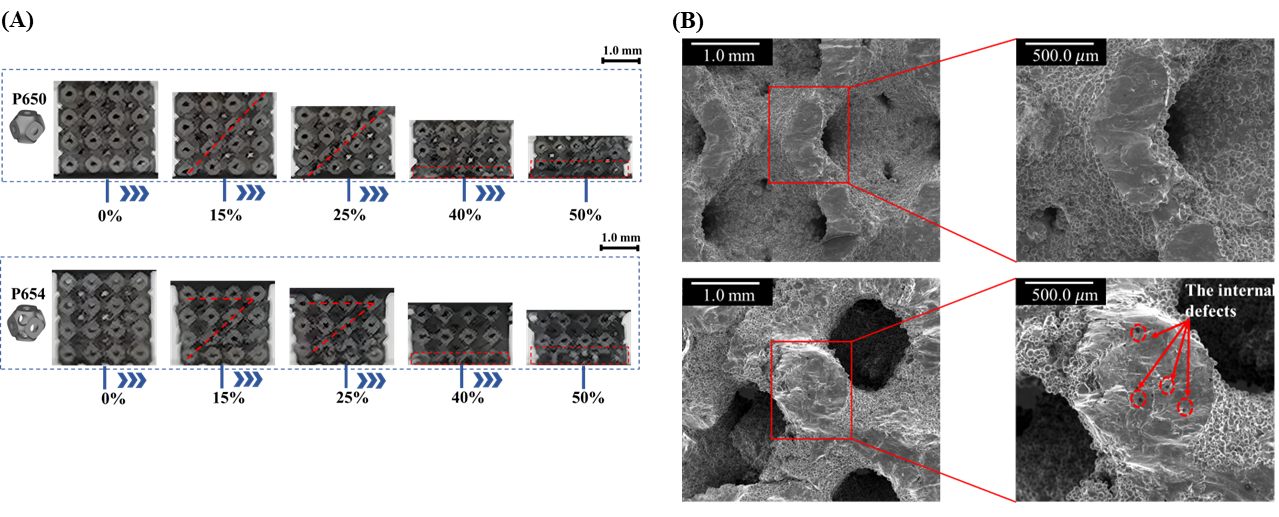

Supplement: Supplementary file 1 [file DataSheet1.ZIP › Supplementary Material Presentation/Figure 3.4.png]

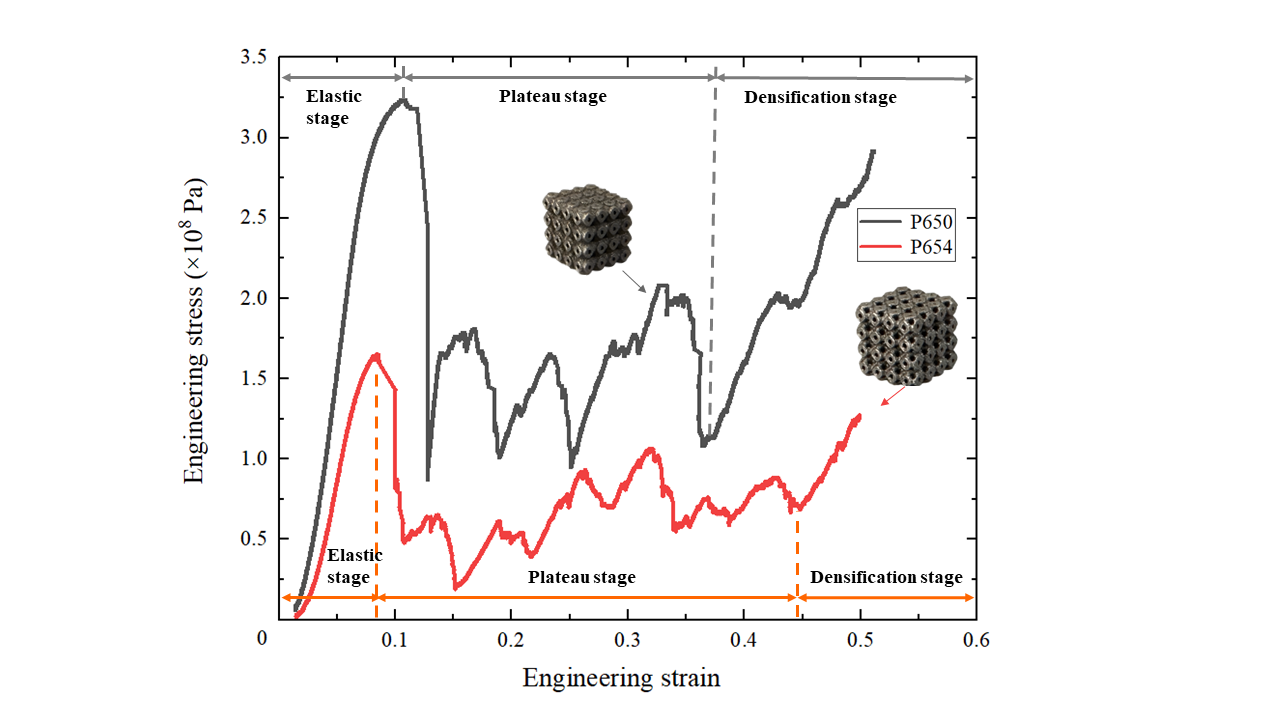

Supplement: Supplementary file 1 [file DataSheet1.ZIP › Supplementary Material Presentation/Figure 3.5.png]

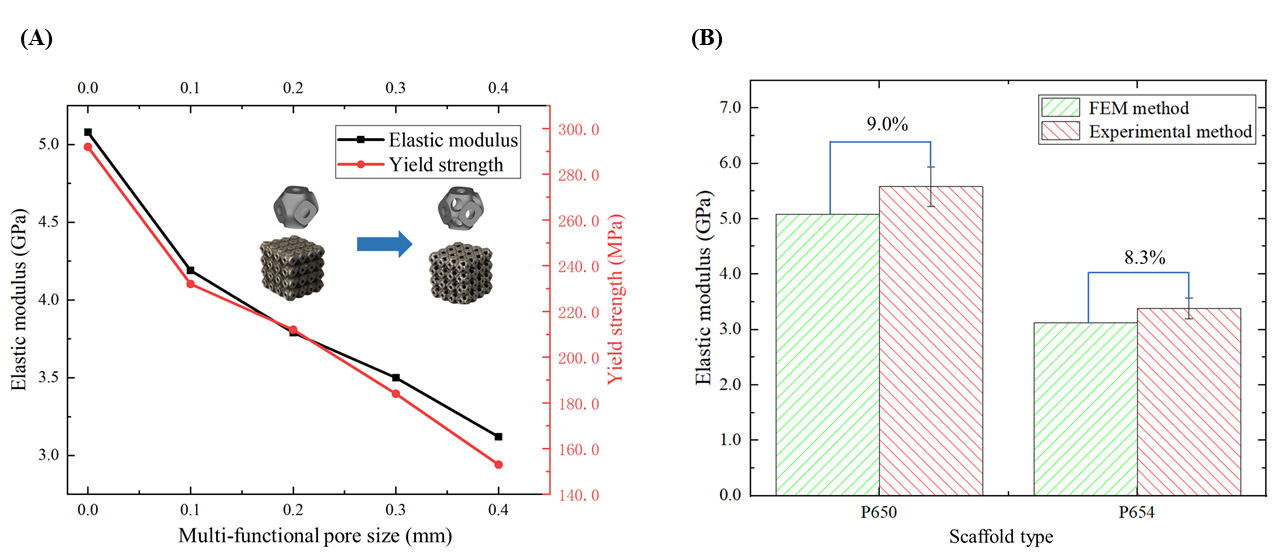

Supplement: Supplementary file 1 [file DataSheet1.ZIP › Supplementary Material Presentation/Figure 3.6.png]

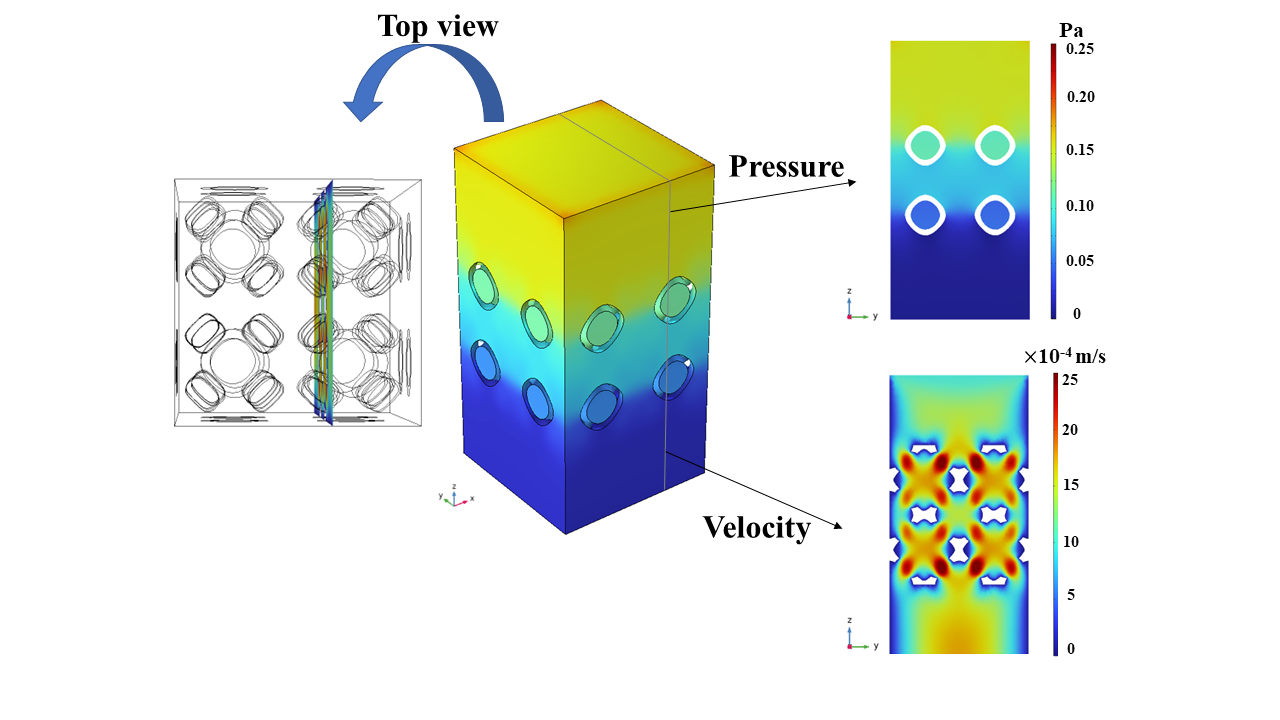

Supplement: Supplementary file 1 [file DataSheet1.ZIP › Supplementary Material Presentation/Figure 3.7.png]

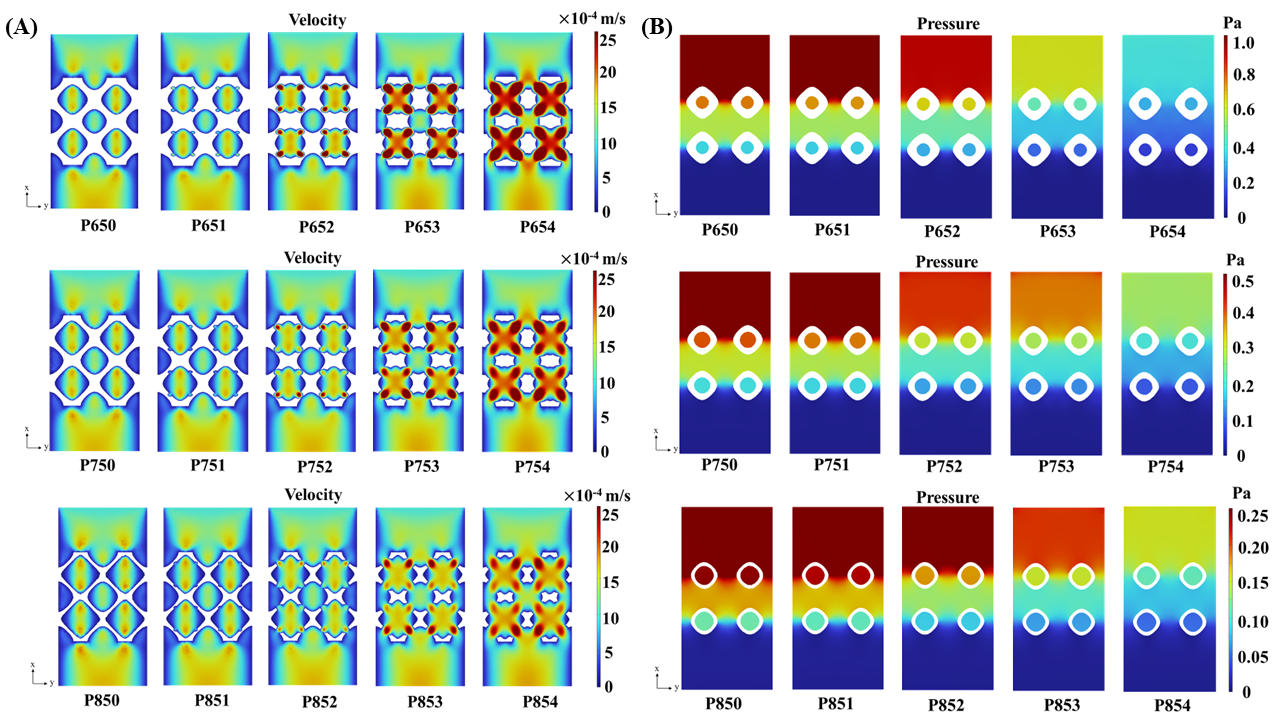

Supplement: Supplementary file 1 [file DataSheet1.ZIP › Supplementary Material Presentation/Figure 3.8.png]

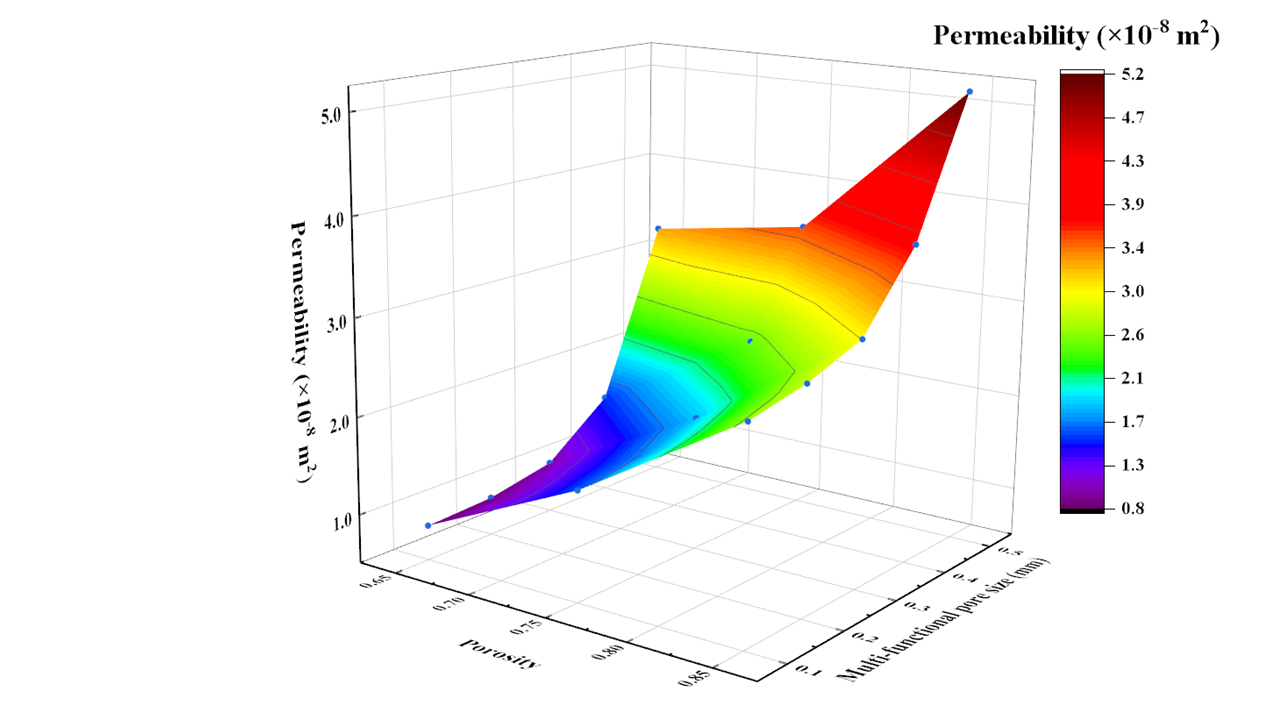

Supplement: Supplementary file 1 [file DataSheet1.ZIP › Supplementary Material Presentation/Figure 3.9.png]
